# Pharmacological Effects and Molecular Mechanisms of Lignans in the Treatment of Alzheimer’s Disease

**DOI:** 10.3390/molecules31152594

**Published:** 2026-07-24

**Authors:** Limeng Sun, Xingyu Zhu, Chunyan Fan, Yu Wang, Qingshan Chen, Lili Zhang, Yiqiang Zhang, Yan Liu

**Affiliations:** 1 Key Laboratory of Basic and Application Research of Beiyao (Heilongjiang University of Chinese Medicine), Ministry of Education, Harbin 150040, China; 2 Traditional Chinese Medicine Biological Genetics, Heilongjiang Province Double First-Class Construction Interdiscipline, Harbin 150040, China; 3 College of Agriculture, Northeast Agricultural University, Harbin 150030, China

**Keywords:** lignans, Alzheimer’s disease, neuroprotection, molecular mechanisms, signaling pathways

## Abstract

Alzheimer’s disease (AD) is a complex, multifactorial neurodegenerative disorder whose core pathological hallmarks include Aβ aggregation, tau hyperphosphorylation, chronic neuroinflammation, oxidative stress, mitochondrial dysfunction, and gut microbiota dysbiosis. Lignans, a class of naturally occurring polyphenolic dimers widely distributed in medicinal plants and diet, exhibit multi-target neuroprotective effects with low toxicity. This review provides a systematic synthesis of the anti-AD pharmacological mechanisms underlying nine structurally distinct lignan subtypes—dibenzocyclooctadiene, tetrahydrofuran, bisepoxy, benzofuran, and biphenyl types—emphasizing scaffold-dependent structure–activity relationships. Key mechanistic pathways encompass direct inhibition of Aβ aggregation and tau phosphorylation, activation of the Nrf2 antioxidant signaling axis and PI3K/Akt pro-survival pathways, suppression of NF-κB-mediated neuroinflammation, restoration of cholinergic function, protection of mitochondria via SIRT3, inhibition of ferroptosis through Gsk3β/Nrf2/GPX4 signaling, and modulation of the gut–brain axis via microbiota-mediated conversion to enterolactone. This review addresses key pharmacokinetic limitations such as low oral bioavailability, rapid metabolism, and limited brain exposure, alongside strategies including structural modification, brain-targeted delivery systems, and gut microbiota modulation. Despite promising preclinical evidence, clinical translation remains limited. Future research priorities should focus on direct target validation, network pharmacology, optimized formulations, and well-designed clinical trials to develop lignans into next-generation anti-aging and anti-AD therapeutics.

## 1. Introduction

Dementia represents a rapidly escalating global public health priority, with advancing age constituting the strongest non-modifiable risk factor. In 2021, the number of individuals living with dementia worldwide reached 57 million, of whom over 60% were in low- and middle-income countries; meanwhile, nearly 10 million new cases occur each year, making prevention and control increasingly urgent [1]. According to the World Health Organization (WHO), both the number and proportion of the global population aged 60 and over are continuously rising. In 2019, this cohort comprised approximately 1 billion individuals; it is projected to reach to 1.4 billion by 2030, and exceed 2.1 billion by 2050. This process of population aging is progressing at an unprecedented pace and will continue to accelerate in the coming decades, particularly in developing countries. The accelerating pace of population aging directly contributes to the escalating global prevalence of dementia. Alzheimer’s disease (AD) is the most prevalent dementia subtype; it exhibits epidemiological patterns that closely mirror those of dementia overall, thereby reinforcing its designation as a leading global health priority requiring urgent, rigorously evidence-based intervention. As a chronic progressive neurodegenerative disease, Alzheimer’s disease typically presents with memory loss as the initial symptom, follows a progressive course, and can ultimately lead to death. Its incidence increases significantly with age, making it one of the high-risk diseases that threaten human health [2]. Currently, no disease-modifying therapy exists for AD, and patients require long-term medication after diagnosis to delay disease progression, imposing a heavy disease burden on most countries and regions worldwide. Notably, women and the very elderly are at relatively higher risk and should be the key focus of clinical attention and prevention and control efforts. Against this backdrop, developing more effective prevention strategies and therapeutic interventions has become an urgent priority for reducing the disease burden of dementia [3].

AD brains exhibit show accumulation of amyloid-β (Aβ) plaques and neurofibrillary tangles (NFTs), along with neuroinflammation, synaptic dysfunction, mitochondrial and bioenergetic disturbances, and vascular abnormalities. Collectively, these processes can ultimately lead to neuronal death [4,5]. AD presents with a range of clinical symptoms; early-stage symptoms are predominantly amnestic, featuring prominent impairment in episodic memory and delayed recall. With the progression of AD, affected individuals may develop deficits in complex attention, expressive language, visuospatial abilities, and executive function [6]. Common AD comorbidities include dyslipidemia, hypertension, diabetes mellitus, obesity, depression, and cardiovascular disease. Complications arising from the progression of AD, such as thrombosis, mobility impairment, dysphagia, malnutrition, and pulmonary infection, can reduce patients’ quality of life and increase the risk of death [7,8,9,10,11,12]. Therefore, a large number of researchers have turned their attention to AD. A growing body of evidence indicates that immune processes play a key role in the pathogenesis of AD [13]. AD reflects systemic dysregulation of core aging processes: mitochondrial dysfunction, oxidative stress, neuroinflammation, ferroptosis, gut–brain axis disruption, and pathogen-associated molecular patterns. These mechanisms synergistically amplify Aβ and Tau pathology, driving progressive cognitive decline. Current clinical drugs can only alleviate symptoms and have safety limitations; therefore, there is an urgent clinical need for natural multi-target molecules that can simultaneously target aging and AD. In the past, lignans and their extracts have been reported to effectively protect neuronal cells and improve cognitive function [14]. Among the various classes of natural products, lignans, a group of polyphenolic dimers generated through oxidative coupling of two phenylpropanoid units has emerged as particularly interesting candidates. Earlier studies have shown that lignans and their crude extracts can protect neuronal cells and improve cognitive performance in experimental models [15]. Lignans align well with the demands of AD drug development. On the one hand, their moderate lipophilicity and relatively low molecular weight facilitate passage across the blood–brain barrier, a prerequisite for central nervous system activity. On the other hand, they display considerable structural variety, encompassing dibenzocyclooctadiene, tetrahydrofuran, bisepoxy, benzofuran, and biphenyl skeletons, which enables them to engage multiple AD-related targets, including Aβ aggregation, tau hyperphosphorylation, oxidative stress, neuroinflammation, and mitochondrial dysfunction. In addition, many lignans are found in edible or medicinal plants and show favorable safety profiles, making them attractive for long-term use as preventive or adjunctive agents. These attributes compare favorably with those of other natural product classes. The bisepoxy type functions as a natural prodrug, undergoing gut microbial conversion to active metabolites like enterolactone. This provides an additional layer of regulation through the gut–brain axis, a feature rarely seen in other classes of phytochemicals. Several earlier reviews have examined the neuroprotective effects of lignans, but most concentrate on individual compounds or narrowly defined mechanisms, with limited attention to systematic structure–activity relationships (SARs) across different lignan subtypes. In addition, emerging topics such as ferroptosis, SIRT3-dependent mitochondrial regulation, and microbiota–gut–brain crosstalk have received little coverage in the existing literature. There is also a notable absence of an aging-centered conceptual framework that explicitly connects the anti-aging properties of lignans with their potential against AD. The present review aims to fill these gaps. We offer a systematic integration of lignans’ multi-target mechanisms from the perspective of aging-driven AD pathology, construct a scaffold–activity–target network that covers the major lignan subtypes, and discuss several cutting-edge topics—ferroptosis, SIRT3, the gut–brain axis, and pathogen infection—that have not been comprehensively addressed before. Our overarching goal is to strengthen the conceptual foundation, innovative potential, and translational relevance of lignan research, focusing on the long-term objective of developing dual-function agents that counteract both aging and Alzheimer’s disease.

### 1.1. Literature Search Strategy

A systematic literature search was conducted across multiple electronic databases, (PubMed, Google Scholar, Sci-Hub, and CNKI) to retrieve publications concerning lignans for the treatment of Alzheimer’s disease from 1990 to 2025. The search strategy employed key terms including: “lignans”, “Alzheimer’s disease”, “neuroprotection”, “amyloid-β”, “tau”, “oxidative stress”, “neuroinflammation”, “cholinergic system”, “ferroptosis”, “gut-brain axis”, “pharmacokinetics” and “structure-activity relationship”.

### 1.2. The Major Pathological Features and Hypotheses of Alzheimer’s Disease

Under physiological conditions, amyloid precursor protein (APP) is processed via the secretory pathway: it moves from the endoplasmic reticulum to the Golgi apparatus, and is subsequently delivered to the cell surface, where it undergoes proteolytic modification. The canonical non-amyloidogenic pathway proceeds as follows: α-secretase first cleaves APP to generate the soluble sAPPα protein; the remaining protein fragment is subsequently processed by γ-secretase, ultimately producing the APP intracellular domain (AICD) and the p3 peptide. In contrast, under pathological conditions, APP enters an alternative metabolic pathway—the endosomal lysosomal hydrolytic pathway. There, sequential cleavage by β-secretase and γ-secretase occurs: β-secretase cleaves APP within the luminal domain of endosomes, while γ-secretase acts on the C-terminal region of APP that is embedded in the cytoplasm. Cleavage of APP by β-secretases releases soluble APPβ protein; subsequent hydrolysis by γ-secretase generates, in addition to AICD, an insoluble peptide known as Aβ. Studies have shown that in patients with AD, the frequency of abnormal APP cleavage via the β-secretase pathway is 50% higher than that in healthy individuals. This alteration directly results in a significant elevation in extracellular Aβ levels. Excessive Aβ aggregates bind apolipoprotein E (APOE), induce axonal degeneration, and activate microglia and astrocytes—triggering sustained neuroinflammation via pro-inflammatory cytokine release—and thereby drive AD [16,17]. The core neuropathological hallmarks of AD derive from the progressive extracellular accumulation of Aβ and the intraneuronal accumulation deposition of hyperphosphorylated tau protein. The massively accumulated Aβ aggregates and deposits to form characteristic amyloid plaques, while hyperphosphorylated tau undergoes conformational changes and assembles into neurofibrillary tangles (NFTs); together, these two lesions constitute the hallmark pathologies for the pathological diagnosis of AD [18,19,20].

#### 1.2.1. The β-Amyloid Hypothesis

The amyloid cascade hypothesis posits that the onset of Alzheimer’s disease begins with the abnormal deposition of Aβ in the brain. Amyloid precursor protein (APP) in the brain, when subjected to abnormal cleavage by β- and γ-secretases, produces highly adhesive Aβ fragments, among which Aβ_42_ is the most pathogenic. These Aβ fragments first aggregate to form soluble oligomers and eventually further deposit as insoluble amyloid plaques [21]. This process is widely regarded as the “initiating event” and “core driver” of AD, although there are subsequent pathological and clinical features—including neurofibrillary tangles formed by tau hyperphosphorylation, neuronal apoptosis, and cognitive decline [22]. Notably, amyloid plaque formation of amyloid plaques is essentially the result of continuous accumulation and progressive aggregation of Aβ monomers in the brain due to overproduction; meanwhile, the progression of cognitive impairment is multifactorial, and the factors include inflammatory responses, abnormal microglial activation, and Aβ aggregation. Furthermore, acetylcholinesterase (AChE) can bind to Aβ to form AChE–Aβ complexes, which further induce the aggregation and deposition of insoluble Aβ fibrils, thereby accelerating the progression of cognitive impairment [21,23].

#### 1.2.2. The Tau Protein Hypothesis

Tau is a microtubule-associated protein (MAP) primarily concentrated in neuronal axons. In AD and related tauopathies, it aggregates to form insoluble filaments and further deposits as NFTs [24]. Under physiological conditions, tau stabilizes microtubules and binds nucleic acids to support genomic and transcriptomic integrity [25]. In AD brain tissue, kinase overactivation and phosphatase loss drive tau hyperphosphorylation, leading to paired helical filament formation and insoluble neurofibrillary tangle (NFT) accumulation, disrupting synaptic plasticity and causing cognitive dysfunction [26]. Tau may contribute to neuronal excitotoxicity [27]. Tau protein in the synapses of healthy brains suggests its involvement in the regulation of synaptic function; meanwhile, the extensive synapse loss observed in the brains of patients with tauopathies may be partially attributed to enhanced toxic effects of tau protein at synaptic sites [28].

#### 1.2.3. Neuroinflammation

Substantial evidence suggests that neuroinflammation is a key participant in the pathological progression of AD. When activated by neurodegenerative changes or neuronal dysfunction, neuroinflammation not only accelerates disease progression but also drives it toward chronicity. In the brain tissue of AD patients, microglia, astrocytes, and neurons express and release various neuroinflammatory mediators, encompassing multiple categories including complement activators and inhibitors, chemokines, cytokines, reactive oxygen species, and inflammatory enzyme systems [29]. Notably, inflammatory cytokines, represented by tumor necrosis factor-alpha (TNF-α) and interleukin-6 (IL-6), directly exacerbate Aβ production in the brain by upregulating β-site amyloid precursor protein cleaving enzyme 1 (BACE1) activity and nuclear factor kappa-B (NF-κB) expression, thereby forming a vicious cycle of “inflammation–Aβ deposition” [30]. Among these, microglia and astrocytes constitute the principal sources of inflammatory cytokines in the AD brain [31]. The aberrant activation of both, together with the cytokines they secrete and the dysregulation of downstream immune signaling pathways, collectively induces pathological neuroinflammatory responses, which, in turn, trigger oxidative stress damage and toxic effects on neurons, thereby driving the continued progression of AD. Given the central role of neuroinflammation in the pathological network of AD, targeted modulation of the initiation and progression of neuroinflammation has become a highly promising research direction in the field of AD treatment, with strong translational potential [32].

#### 1.2.4. The Cholinergic Hypothesis

Acetylcholine (ACh) is a critical excitatory neurotransmitter in the central nervous system, essential for learning, memory consolidation, attention, and other higher cognitive functions. Its levels in the brain are primarily maintained by the central cholinergic system through the regulation of acetylcholine synthesis and release [33]. In AD brain tissue, dysregulation of multiple neurotransmitter systems is consistently observed, including decreases in acetylcholine, norepinephrine, and serotonin, with acetylcholine deficiency being the most pronounced. This characteristic biochemical alteration directly prompted the formulation of the cholinergic hypothesis of AD, which proposes that the memory decline and cognitive abnormalities seen in AD patients are largely attributable to the reduction in acetylcholine in the brain [34,35]. Clinical evidence has confirmed that the cognitive impairments in AD, such as typical symptoms like memory loss, are closely associated with the progressive dysfunction of central cholinergic and glutamatergic neurotransmission [36]. The severity of cognitive deficits is also positively correlated with the degeneration of cholinergic neurons [37]. Thus, dysfunction of the cholinergic system plays a key role in the development and progression of cognitive impairment in AD [38]. Persistent deficiency of acetylcholine in the brain leads to a progressive and significant decline in cognitive and behavioral functions in patients [39]. Moreover, central cholinergic dysfunction contributes to AD pathogenesis through multiple convergent pathways, including aberrant tau phosphorylation, neuroinflammatory activation, and neuronal apoptosis, thereby further aggravating disease progression [40].

#### 1.2.5. Oxidative Stress

AD is associated with multiple etiologies and pathological mechanisms, among which oxidative stress (OS) is considered one of the key core factors regulating the onset and progression of the disease. Oxidative stress is defined as “an imbalance between pro-oxidants and antioxidants, accompanied by disruption of redox circuits and macromolecular damage” [41]. OS disrupts multiple critical neurobiological pathways involved in the regulation of brain function [42]. OS is considered a bridge connecting the different hypotheses and mechanisms of AD [43]. Elevated levels of reactive oxygen species (ROS), generated by the abnormal accumulation of Aβ peptides, further exacerbate tau pathology, heme dyshomeostasis, mitochondrial dysfunction, and endoplasmic reticulum (ER) stress. Excess ROS can damage essential cellular components [44]. Oxidative stress mediates the neurotoxicity induced by the abnormal accumulation of Aβ and tau, potentially increasing Aβ production and aggregation and promoting tau phosphorylation and neurofibrillary tangles, thereby further enhancing various neurotoxic events, including ROS production, and thus forming a vicious cycle that promotes the onset and progression of AD [45].

#### 1.2.6. Mitochondrial Dysfunction

Mitochondria are evolutionarily conserved, multifunctional organelles central to cellular bioenergetics, including ATP synthesis, calcium homeostasis, and cell survival and death. At the same time, the mitochondrial respiratory chain is the major site of ROS production in cells, and mitochondria are particularly vulnerable to oxidative stress [46,47]. Extensive studies have demonstrated that mitochondrial dysfunction is an important factor involved in the pathogenesis of AD. Mitochondrial abnormalities are accompanied by oxidative damage marked by 8-hydroxyguanosine and nitrotyrosine, indicating that mitochondria are damaged during the progression of AD [48]. It has been reported that the activity of mitochondrial cytochrome oxidase (complex IV) is significantly reduced in the cortical regions of the AD brain. Deficiency of this key electron transport enzyme may lead to increased ROS production and reduced energy stores, collectively accelerating synaptic degeneration and neuronal loss in AD [49]. In the brain, given the extremely high bioenergetic demands of neurons to sustain synaptic activity and plasticity, mitochondrial abnormalities are among the earliest detectable changes in the pathology of AD [50]. Multiple interrelated factors, including metabolic dysregulation, oxidative stress, disrupted calcium homeostasis, and impaired mitochondrial quality control, are associated with the mitochondrial dysfunction observed in AD [51].

#### 1.2.7. Gut Microbiota

Gut microbiome dysbiosis accelerates late-stage AD progression, manifested by aggravated cognitive deficits, neuroinflammation, and Aβ/hyperphosphorylated tau aggregation. These results confirm the gut–brain axis as a key regulatory hub in AD and identify the microbiome as a promising therapeutic target [52]. Gut dysbiosis in AD increased intestinal permeability, enabling microbial metabolites and bacteria to enter the bloodstream, thereby activating immune cells. This drives neuroinflammation, neuronal loss, and cognitive decline. Notably, colonization of Porphyromonas gingivalis within the central nervous system also induces enhanced neuroinflammatory responses and abnormal accumulation of Aβ [53]. There is continuous bidirectional communication and mutual regulation between the gastrointestinal tract and the central nervous system, mediated by the gut–brain axis. A large body of research has confirmed that changes in the composition of the gut microbiota are closely associated with the progression of AD, and that restoring a healthy gut microbiota homeostasis holds promise for delaying or even ameliorating the clinical symptoms and pathological progression of AD. Therefore, targeted modulation of the gut microbiota has emerged as a novel paradigm in the clinical management of AD, and extensive current research is dedicated to developing new preventive and therapeutic strategies for AD based on this target [54].

**Figure 1:**
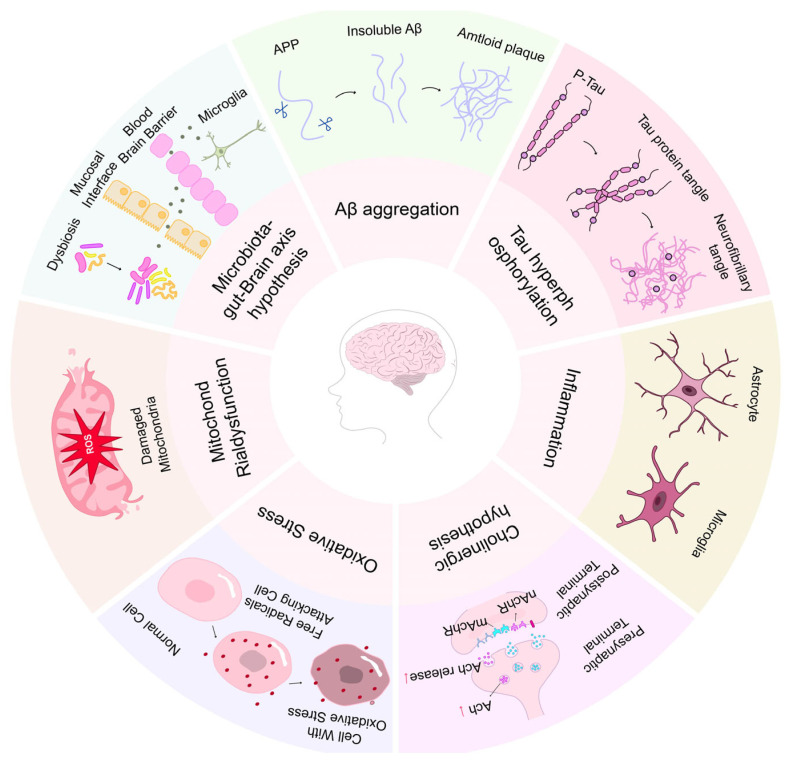
Schematic diagram of pathological mechanisms of Alzheimer’s disease.

Alzheimer’s disease stands as a prototypical neurodegenerative illness closely linked to aging. Its onset does not stem from one solitary pathological pathway; instead, it develops through a stepwise pathological cascade where different pathological events reinforce one another. The abnormal aggregation of amyloid-beta peptides and hyperphosphorylation of tau protein sit at the heart of this pathological network, two pathological changes generally recognized as the leading triggers behind disease progression. As these two core pathological features build up in the brain, they set off multiple subsequent pathological responses, such as chronic neuroinflammation, oxidative stress and cholinergic signaling. These secondary pathological changes will, in turn, further worsen the primary pathological lesions. Under this pathological model, newly discovered pathological pathways including ferroptosis and intestinal flora imbalance mainly act as key downstream regulatory mediators and peripheral risk amplifiers, instead of standalone core drivers AD. Via the gut–brain axis, these factors accelerate neuronal degeneration and exacerbate Aβ- and tau-mediated neuropathology, establishing a self-perpetuating cycle that drives progressive disease advancement. Collectively, these interconnected pathological processes culminate in synaptic loss, irreversible neuronal apoptosis, and progressive cognitive decline, constituting the core age-dependent pathogenic architecture of AD (Figure 1).

## 2. Overview of Lignans

Lignans are plant-derived secondary metabolites ubiquitous in the human diet. Structurally, they consist of two phenylpropane (C6–C3) units linked via A β, and β-bond, with variations in the degree of side-chain oxidation and diverse substitution patterns on the benzene rings. Traditionally, lignans are divided into two major groups: classical lignans and neolignans. Lignans function as phytochemical defense compounds, conferring resistance to microbial pathogens in plants [55,56,57]. In addition, dietary lignan intake helps reduce the risk of cancer [58,59]. Over the course of extensive research, lignans have been shown to exhibit a wide range of pharmacological activities [60]. These include antibacterial [61], antiviral [62], antitumor [63,64,65], antiplatelet [66], phosphodiesterase-inhibitory [67,68], cytotoxic [69], antioxidant [70] and immunosuppressive [71] activities. Studies have demonstrated that certain lignans can effectively inhibit the aggregation of Aβ, the abnormal deposition of which is one of the core pathological hallmarks of AD. Meanwhile, the antioxidant and anti-inflammatory properties of lignans also make them potential candidates for AD therapy. Therefore, a thorough elucidation of the definition and classification of lignans is of significant theoretical and practical importance for revealing their mechanisms of action in AD treatment.

**Figure 2:**
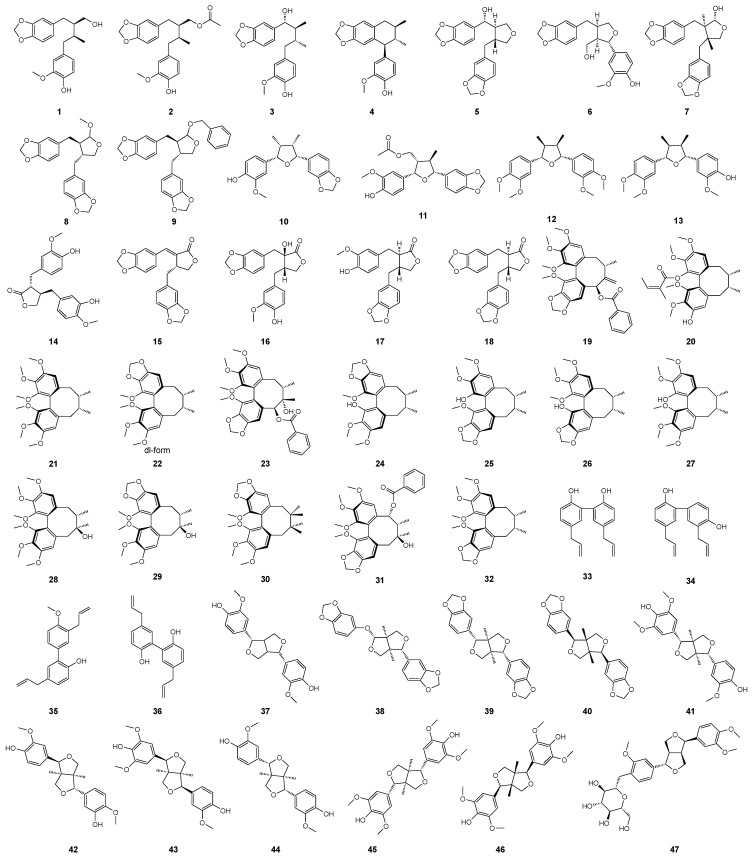

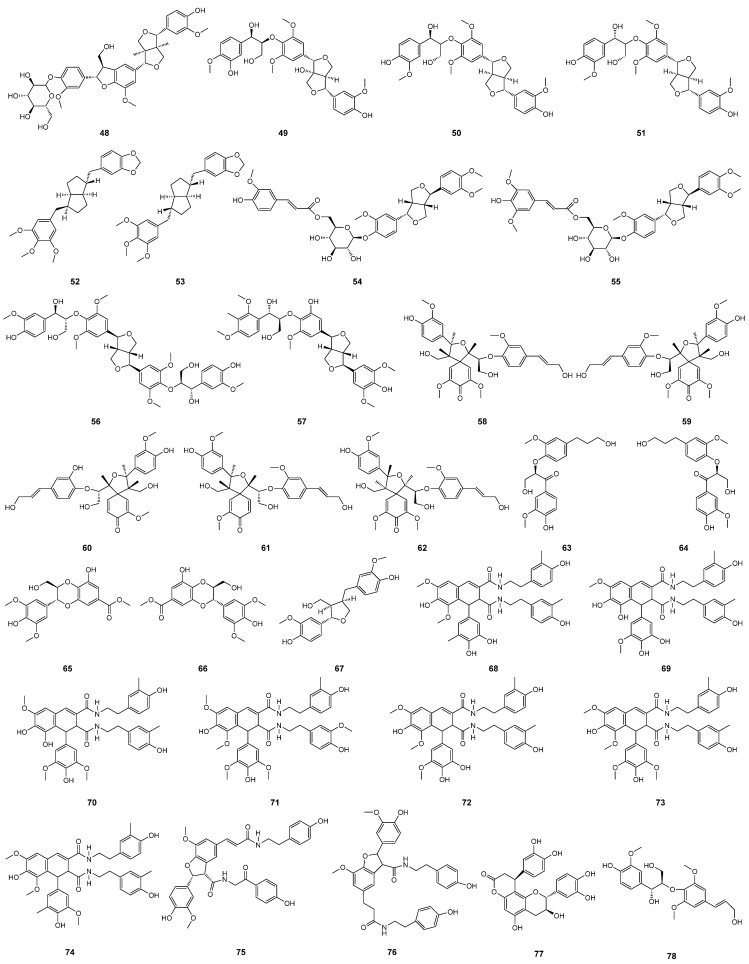

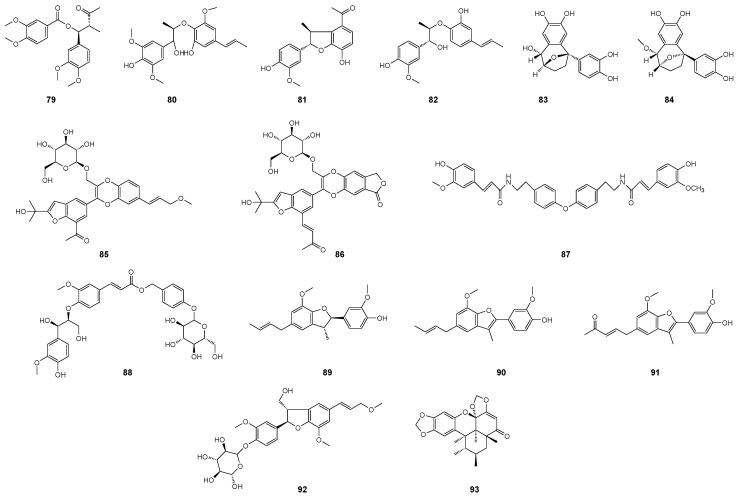
Chemical structures of anti-Alzheimer’s disease lignans (1–93).

Lignans are plant-derived polyphenolic secondary metabolites biosynthesized via oxidative dimerization of phenylpropane (C6–C3) units and are abundant in both medicinal herbs and common dietary sources. Structurally classified according to their carbon skeleton topology, lignans comprise nine principal subtypes: simple lignans (Dibenzylbutane-type lignans), monoepoxylignans (tetrahydrofuran-type lignans), lignanolides, cyclolignans, cyclolignanolides, diepoxylignans, dibenzocyclooctadiene-type lignans, benzofuran lignans, neolignans, and other special types. This paper compiled 93 lignan compounds with protective effects against AD (Table 1), and, for the first time, established a panoramic correspondence rule of the lignan skeleton–AD target-signaling pathway, clarifying that different skeleton structures directly determine their lipophilicity, blood–brain barrier penetration ability, and anti-AD action preference: dibenzocyclooctadiene-type lignans, such as gomisin N, schisandrin A, and schisandrin B, can selectively modulate the Nrf2/GSK-3β/SIRT3 signaling pathway. This effect has been observed in both cellular assays and rodent models of AD, providing a mechanistic basis for their antioxidant activity and mitochondrial protection. Notwithstanding these promising findings, the majority of the supporting evidence derives from in vitro systems, with only a limited number of in vivo studies available, and direct target binding has yet to be conclusively demonstrated. Several tetrahydrofuran-type lignans, including cubebin and compounds 58–61 from *Isatis indigotica*, have shown acetylcholinesterase inhibitory activity in enzymatic assays. Nevertheless, whether these in vitro observations translate into meaningful cognitive benefits in vivo remains unestablished and warrants further exploration. Bisepoxy-type lignans appear to function as natural prodrugs, requiring gut microbiota-mediated conversion into enterolactone to exert their effects. Biphenyl-type lignans, on the other hand, have been linked to the activation of the AMPK/mTOR/ULK1 pathway, which may promote autophagy and facilitate Aβ clearance. For benzofuran-type lignans, preliminary evidence suggests activity against ferroptosis-related pathways in cellular models, although the data are still limited and in vivo corroboration is needed. Additional activities, including inhibition of Tau hyperphosphorylation, anti-inflammatory effects, and gut microbiota modulation, have also been reported for various lignan subtypes. Collectively, these findings establish a foundational structure–activity relationship (SAR) framework that informs rational ligand screening, scaffold-based structural optimization, and hypothesis-driven mechanistic studies of lignans for Alzheimer’s disease intervention. The pathogeneses of AD in this paper are shown in Figure 2.

**Table 1:** Sources of lignans with anti-Alzheimer’s disease activity.

No.	Name	Family	Source	Tissue	Reference
1	Schibitubin C	Schisandraceae	*Schisandra* *bicolor* var. *tuberculata*	Fruits	[72]
2	Schibitubin D	Schisandraceae	*Schisandra bicolo* var. *tuberculata*	Fruits	[72]
3	Oleiferin-F	Schisandraceae	*Schisandra bicolo* var. *tuberculata*	Fruits	[72]
4	(−)-Isootobaphenol	Schisandraceae	*Schisandra bicolo* var. *tuberculata*	Fruits	[72]
5	Isocubebin	Thymelaeaceae	*Wikstroemia alternifolia*	Branches/Leaves	[73]
6	Sanshodiol	Thymelaeaceae	*Wikstroemia alternifolia*	Branches/Leaves	[73]
7	(−)-Cubebin	Piperaceae	*Piper cubeba*	Seeds	[74]
8	(−)-O-Methylcubebin	The synthesized compounds	/	/	[74]
9	(−)-O-Benzylcubebin	The synthesized compounds	/	/	[74]
10	(−)-Talaumidin	Aristolochiaceae	*Aristolochia arcuata* Masters	Roots	[75]
11	Schibitubin H	Schisandraceae	*Schisandra bicolo* var. *tuberculata*	Fruits	[72]
12	Galgravin	Schisandraceae	*Schisandra bicolo* var. *tuberculata*	Fruits	[72]
13	(−)-Nectandrin-A	Schisandraceae	*Schisandra bicolo* var. *tuberculata*	Fruits	[72]
14	Arctigenin	Asteraceae Asteraceae Convolvulaceae Asteraceae Taxaceae	*Arctium lappa* L. *Bardanae Fructus* *Ipomoea cairica* (L.) *Sweet* *Saussurea medusa Maxim.* *Torreya nucifera* (L.) *Siebold & Zucc.*	Fruits Fruits Fruits Fruits Fruits	[76]
15	Savinin	Araliaceae	*Eleutherococcus henryi Oliv.*	Roots	[77]
16	Wikstralternifols and B	Thymelaeaceae	*Wikstroemia alternifolia*	Branches/Leaves	[73]
17	Pluviatolide	Thymelaeaceae	*Wikstroemia alternifolia*	Branches/Leaves	[73]
18	Hinokinin	Thymelaeaceae	*Wikstroemia alternifolia*	Branches/Leaves	[73]
19	Schisanchinin A	Schisandraceae	*Schisandra chinensis*	Fruits	[78]
20	Schisanchinin B	Schisandraceae	*Schisandra chinensis*	Fruits	[78]
21	Deoxyschizandrin	Schisandraceae	*Schisandra chinensis*	Fruits	[78]
22	(±)-γ-Schizandrin	Schisandraceae	*Schisandra chinensis*	Fruits	[78]
23	Gomisin G	Schisandraceae	*Schisandra chinensis*	Fruits	[78]
24	(−)-Gomisin M1	Schisandraceae	*Schisandra chinensis*	Fruits	[78]
25	(−)-Gomisin L1	Schisandraceae	*Schisandra chinensis*	Fruits	[78]
26	(+)-Gomisin M2	Schisandraceae	*Schisandra chinensis*	Fruits	[78]
27	(+)-Gomisin K3	Schisandraceae	*Schisandra chinensis*	Fruits	[78]
28	Schisandrin	Schisandraceae	*Schisandra chinensis*	Fruits	[78]
29	Gomisin A	Schisandraceae	*Schisandra chinensis*	Fruits	[78]
30	Gomisin N	Schisandraceae	*Schisandra chinensis*	Fruits	[78]
31	Schisantherin A	Schisandraceae	*Schisandra chinensis*	Fruits	[79]
32	Schisandrin B	Schisandraceae	*Schisandra chinensis*	Fruits	[80]
33	Magnolol	Magnoliaceae	*Magnolia officinalis*	Barks	[81]
34	Honokiol	Magnoliaceae	*Magnolia officinalis*	Barks	[82]
35	4-O-methylhonokiol	Magnoliaceae	*Magnolia officinalis*	Barks	[83]
36	Obovatol	Magnoliaceae	*Magnolia officinalis*	Baeks	[83]
37	Pinoresinol	Eucommiaceae	*Eucommia ulmoides Oliver*	Leaves	[84]
38	Sesamolin	Pedaliaceae	*Sesamum indicum*	Seeds	[85]
39	Sesamin	Pedaliaceae	*Sesamum indicum*	Seeds	[86]
40	(−)-Sesamin	Aristolochiaceae	*Asiasari Radix*	Roots	[87]
41	Medioresinol	Pedaliaceae Rosaceae	*Sesamum indicum* *Cloudberry*	Seeds Fruits	[85]
42	(−)-7-Epi-Pinoresinol Mr1	Eucommiaceae	*Eucommia ulmoides Oliver*	Leaves	[84]
43	(+)-Medioresinol	Eucommiaceae	*Eucommia ulmoides Oliver*	Leaves	[84]
44	(+)-Diapinoresinol	Eucommiaceae	*Eucommia ulmoides Oliver*	Leaves	[84]
45	(+)-Syringaresinol	Araliaceae Rosaceae Magnoliaceae	*Panax ginseng* *Prunus mume* *Magnolia thailandica*	Fruits	[88]
46	Syringaresinol	Poaceae	*Rye, whole Grain flour*	Whole grain	[85]
47	Phillyrin	Oleaceae	*Forsythia suspensa*	Fruits	[89]
48	Anisacanthin	Acanthaceae	*Anisacanthus virgularis Nees*	Aerial Parts	[90]
49	Firmianols B	Eucommiaceae	*Eucommia ulmoides Oliver*	Leaves	[84]
50	Hedyotol C	Eucommiaceae	*Eucommia ulmoides Oliver*	Leaves	[84]
51	Hedyotol D	Eucommiaceae	*Eucommia ulmoides Oliver*	Leaves	[84]
52	Epi-aschantin	Asteraceae	*Artemisia mongolica*	Whole herb	[91]
53	Aschantin	Asteraceae	*Artemisia mongolica*	Whole herb	[91]
54	Aurantiosides C	Oleaceae	*Osmanthus fragrans* var. *aurantiacus*	Leaves	[92]
55	Aurantiosides D	Oleaceae	*Osmanthus fragrans* var. *aurantiacus*	Leaves	[92]
56	Hedyotisol-A	Ranunculaceae	*Aconiti lateralis Radix Praeparata*	Lateral roots	[93]
57	(7*R*,8*R*)-8-syringaresino l-4-hydroxy-3,5-dimethoxyphenyl-7,9-propanediol	Ranunculaceae	*Aconiti lateralis Radix Praeparata*	Lateral roots	[93]
58	Isatispironeol A	Brassicaceae	*Isatis indigotica Fortune*	Leaves/Roots	[94]
59	Isatispironeol A	Brassicaceae	*Isatis indigotica Fortune*	Leaves/Roots	[94]
60	(−)-(7*R*,8*S*,1′*R*,7′*R*,8′*S*)-Sibiricumin A	Brassicaceae	*Isatis indigotica Fortune*	Leaves/Roots	[94]
61	(+)-(7*S*,8*R*,1′*S*,7′*S*,8′*R*)-Sibiricumin A	Brassicaceae	*Isatis indigotica Fortune*	Leaves/Roots	[94]
62	Isatispironeol B	Brassicaceae	*Isatis indigotica Fortune*	Leaves/Roots	[94]
63	(*R*)-1-(3-Methoxy-4-Hydroxyphenyl)-2-(3-Methoxy-1-Hydroxypropylphenoxy)-3-Hydroxypropan	Rosaceae	*Prunus tomentosa Thunb*	Seeds	[95]
64	(*S*)-1-(3-Methoxy-4-Hydroxyphenyl)-2(*T*)-(3-Methoxy-1-Hydroxypropylphenoxy)-3(*U*)-Hydroxypropan	Rosaceae	*Prunus tomentosa Thunb*	Seeds	[95]
65	(7*S*,8*S*)-Pithecellobiumin A	Fabaceae	*Pithecellobium* *clypearia Benth*	Twigs/Leaves	[96]
66	(7*R*,8*R*)-Pithecellobiumin A	Fabaceae	*Pithecellobium* *clypearia Benth*	Twigs/Leaves	[96]
67	(+)-Lariciresinol	Rubiaceae	*Rubia philippinensis*	Roots	[97]
68	1,2-Dihydro-6,8-Dimethoxy-7 -Hydroxy-1-(3,4-Dihydroxyphenyl)-N1N2-Bis-[2-(4-Hydroxyphenyl)Ethyl]-2,3-Naphthalene Dicarboxamide	Monascaceae Annonaceae Solanaceae	*Monascus pilosus* *Porcelia macrocarpa* *Lycium chinense*	Yeast Branchs Roots/barks	[98] [99] [100,101]
69	Methoxynaphthalene-2,3-Dicarboxa mide-1-(3,4-Dihydroxy-5-Methoxyphenyl)-1,2-2-Dihydroxy-6,7-Dihydroxy-N,N′-Bis-[2-(4-Hydroxyphenyl)-Ethyl]-8-Methoxynaphthalene-2,3-Dicarboxamide	Piperaceae Canellaceae	*Piper hancei* *Warburgia ugandensis*	Stems Stems barks	[102] [103]
70	Hancamide C	Piperaceae Commelinaceae Corydalis	*Piper hancei* *Commelina africana* *Saxicola*	Stems Fresh Leaves Aerial parts	[102] [104] [105]
71	Hancamide D	Piperaceae	*Piper hancei*	Stems	[102]
72	Cannabisin A	Moraceae Cannabaceae Aizoaceae	*Cannabis sativa* *Hemp* *Tetragonia tetragonioide*	Fruits/Seeds Seeds Aerial parts	[106,107,108,109] [110] [111]
73	1,2-Dihydro-6,8-Dimethoxy-7-Hydroxy-1-(3,5-Dimethoxy-4-Hydroxyphenyl)-N1,N2-Bis-[2-(4-Hydroxyphenyl)Ethyl]-2,3-Naphthalene Dicarboxamide	Annonaceae Commelinaceae Piperaceae Solanaceae	*Porcelia macrocarpa* *Commelina communis* *Piper flaviflorum* *Solanum melongena*	Branchs Whole plants Aerial parts Roots	[99] [112] [113] [114]
74	Flavifloramide A	Piperaceae Solanaceae	*Piper flaviflorum* *Solanum melongena*	Aerial parts Roots	[113] [114]
75	Tribulusamide B	Zygophyllaceae Annonaceae	*Tribulus terrestris* *Mitrephora thorelii*	Fruits Stems	[115] [116]
76	Grossamide	Moraceae Solanaceae Solanaceae Annonaceae Liliaceae Moraceae Malvaceae Solanaceae	*Ficus foveolata* *Solanum tuberosum* *Withania sominfera* *Xylopia aethiopica* *Smilax scobinicaulis* *Cannabis sativa* *Hibiscus cannabinus* *Solanum melongena*	Stems Scab lesion Fruits Seeds Roots Seeds Barks Roots/the whole plant	[117] [118] [119,120] [121] [122] [106,107,123] [124] [114,125] [115]
		Zygophyllaceae Solanaceae Solanaceae Annonaceae Araceae Commelinaceae	*Tribulus terrestris* *Annuum var. grossum* *Lycium chinense* *Annonacrassiflora* *Alocasia macrorrhiza* *Commelina africana*	Fruits Roots Seeds Seeds Rhizomes Fresh leaves	[126,127] [125] [128] [129] [130]
77	Cinchonain Ib	Eucommiaceae	*Eucommia ulmoides Oliver*	Leaves	[84]
78	threo-guaiacylglycerol-8-O-40-sinapyl alcohol ether	Eucommiaceae	*Eucommia ulmoides Oliver*	Leaves	[84]
79	Schibitubin I	Schisandraceae	*Schisandra bicolo* var. *tuberculata*	Fruits	[72]
80	2-(((1*R*,2*R*)-1-hydroxy-1-(4-hydroxy-3,5-dime-thoxyphenyl)propan-2-yl)oxy)-3-methoxy-5-((*E*)-prop-1-en-1-yl)phenol	Apocynaceae	*Adelostemma gracillimum*	Roots	[131]
81	1-((2*S*,3*S*)-7-hydroxy-2-(4-hydroxy-3-methoxy-phenyl)-3-methyl-2,3-dihydrobenzofuran-4-yl)ethanone	Apocynaceae	*Adelostemma gracillimum*	Roots	[131]
82	Callislignan B	Apocynaceae	*Adelostemma gracillimum*	Roots	[131]
83	Capitugenin A	Hypoxidaceae	*Curculigo capitulata*	Rhizomes	[132]
84	Capitugenin C	Hypoxidaceae	*Curculigo capitulata*	Rhizomes	[132]
85	3,4-(10-methoxy-phenylallyl)-9″-((10′-isopropanol-3′,4′-furan)-phenylacetyl)-8″-dioxane-7″-O-β-D-glucopyranoside	Magnoliaceae	*Magnolia biondii Pamp.*	Flower buds	[133]
86	3,4-benzolactone-9″-((12′-iso- propanol-3′,4′-furan)-phenylbutenone)-8″-dioxane-7″-O-β-D-glucopyranoside	Magnoliaceae	*Magnolia biondii Pamp.*	Flower buds	[133]
87	Lyciumamide A	Solanaceae	*Lycium barbarum*	Fruits	[134,135]
88	Bletineoside C	Orchidaceae	*Bletilla striata* (Thunb.)	Stems	[136]
89	(+)-licarin A	Euphorbiaceae	*Phyllanthodendron breynioides*	Leaves/Twigs	[137]
90	Eupomatenoid-7	Euphorbiaceae	*Phyllanthodendron breynioides*	Leaves/Twigs	[137]
91	Eleganal	Euphorbiaceae	*Phyllanthodendron breynioides*	Leaves/Twigs	[137]
92	Bletineoside D	Orchidaceae	*Bletilla striata* (Thunb.)	Stems	[136]
93	Sauchinone	Saururaceae	*Saururus chinensis*	Whole Grain	[138]

### 2.1. Major Classes of Lignan

Lignans are systematically classified into nine structural subclasses based on carbon–carbon bond connectivity and ring topology of their dimeric phenylpropane core: dibenzocyclooctadiene-type, tetrahydrofuran-type, bisepoxy-type, benzofuran-type, lignanolide-type, amide-type, biphenyl-type, norlignan-type, and other miscellaneous types. Structural divergences include variations in ring architecture and stereochemical configuration; different subtypes exhibit the distinct characteristic that the skeleton determines the activity and the structure determines the target. They can, respectively, target Aβ aggregation, Tau hyperphosphorylation, neuroinflammation, oxidative stress, cholinergic deficit, mitochondrial dysfunction, and gut microbiota dysbiosis, thus constituting a structurally diverse and mechanistically well-defined natural molecular library for the multi-target and multi-pathway prevention and treatment of AD.

The structural differences among the lignan subtypes not only govern their physicochemical properties but also directly influence their binding modes with AD-related targets (such as AChE, Nrf2, and NF-κB). Their multi-target action characteristics are highly compatible with the complex pathological mechanisms of AD, providing a rich molecular structural basis and drug development directions for multi-target AD therapy in the field of geriatric medicine.

### 2.2. Chemical Structures and Source Characteristics of Representative Anti-AD Lignans

The 93 lignans cataloged in Table 1 comprehensively represent all nine structural subtypes (chemical structures shown in Figure 2) and exhibit marked structural diversity in core scaffold architecture, oxygenation patterns, and stereochemical configuration—thereby establishing a rigorous structural foundation for the mechanistic pharmacology analyses presented in Section 3.

Preclinical models have shown that dibenzocyclooctadiene-type lignans, such as schisandrin, gomisin N, and schisandrin B, can penetrate the blood–brain barrier. This capacity appears to arise from their conformationally constrained dibenzocyclooctadiene skeleton, which provides both a well-defined three-dimensional architecture and lipophilicity suitable for BBB passage. Tetrahydrofuran-type lignans, such as cubebin and its analogues from *Isatis indigotica*, feature a saturated tetrahydrofuran ring fused to two aromatic systems. This structure gives them greater conformational flexibility compared to dibenzocyclooctadienes. When hydroxyl and methoxy substituents occupy specific ring positions and are held in stable, favorable conformations, they directly inhibit acetylcholinesterase (AChE). Their potency has been confirmed in standardized enzymatic assays. Bisepoxy-type lignans (such as sesamin, pinoresinol, and syringaresinol) bear two stereochemically defined oxirane rings and are frequently isolated as O-glycosides. Secoisolariciresinol-type lignans exhibit high polarity due to multiple phenolic hydroxyls, necessitating gut microbial deglycosylation and demethylation to yield bioactive enterolactone—a metabolic activation pathway distinguishing them from most other lignan subtypes. Benzofuran-type lignans (including (+)-licarin A and eupomatenoid-7) feature a rigid benzofuran skeleton; structure–activity analyses suggest that a trans-configured C3 side-chain double bond and 4-hydroxy substitution (over 5-hydroxy) correlate with improved neuroprotective efficacy in cellular models, though mechanistic validation is ongoing. Without interphenyl oxygen bridges, biphenyl lignans like magnolol and honokiol have more torsional freedom. This structural feature leads to unique target-binding profiles. Hydroxyl group stereochemistry and regiochemistry critically modulate autophagic flux, although precise molecular targets remain under investigation. Lignanolide-type lignans, including arctigenin and savinin, contain a γ-butyrolactone ring. In the case of arctigenin, removing the sugar group through enzymatic or microbial deglycosylation generates the aglycone, which consistently shows stronger neuroprotective and anti-inflammatory effects in cell-based assays. Glycosylation status therefore emerges as a crucial factor governing both the pharmacokinetics and pharmacodynamics of these compounds. Lignan amides such as cannabisin and lyciumamide A contain amide bonds, which give them an amphiphilic nature. In activated microglia, they can selectively dampen pro-inflammatory pathways, including NF-κB and NLRP3 signaling. Dibenzylbutane-type lignans, including schibitubin C and galgravin, are characterized by a C8–C8′ bond connecting two phenylpropane units. Their radical-scavenging ability largely depends on the number and positioning of phenolic hydroxyl, methoxy, and methylenedioxy groups. Although norlignans and other structurally atypical lignan subtypes are chemically diverse, they have received little pharmacological attention. To date, very little in vitro or in vivo activity data have been reported for these compounds.

Collectively, these structural distinctions establish a chemically grounded framework for rationalizing scaffold-dependent lignan bioactivities. Current structure–activity correlations, however, are largely drawn from narrow chemical series and early-stage experimental data. They should therefore be seen as provisional working hypotheses, pending further refinement through broader SAR profiling, target engagement validation, and functional phenotypic confirmation. Rigorous mechanistic analyses for each lignan subtype are detailed in Section 3.

## 3. Pharmacological Effects and Molecular Mechanisms of Lignans in the Treatment of Alzheimer’s Disease

With their unique advantages of multi-target action, low toxicity, blood–brain barrier permeability, and anti-aging protective effects, lignans can systematically intervene in the aging-driven cascade pathological network of AD, from inhibiting Aβ deposition, antagonizing Tau hyperphosphorylation, counteracting oxidative stress, suppressing neuroinflammation, restoring the cholinergic system, protecting mitochondrial function, and inhibiting ferroptosis to modulating the gut–brain axis, thereby achieving comprehensive neuroprotection. Their pharmacological effects are not exerted through isolated target binding; rather, they form a synergistic regulatory network via core signaling hubs such as Nrf2, NF-κB, PI3K/Akt, AMPK, SIRT3, and GSK-3β, ultimately disrupting the vicious pathological cycle, rescuing synaptic function, and ameliorating cognitive impairment.

### 3.1. Targeting β-Amyloid Aggregation and Clearance

The abnormal production and aggregation of Aβ are generally considered to be important events in the early stages of AD. In various experimental systems—ranging from cell-free assays to transgenic mice—misfolded Aβ monomers have been shown to form oligomers and fibrils, and these species have been associated with oxidative stress, inflammatory responses, and neuronal dysfunction. A number of lignans have been examined for their ability to interfere with this process, and the available data, though uneven in quality and quantity, point to several possible mechanisms. The most direct evidence comes from cell-free enzymatic assays (e.g., ThT fluorescence), where certain lignans have been shown to bind Aβ and inhibit fibril formation. Cell-based studies have provided additional observations, including suppression of BACE1 activity and enhancement in autophagic clearance, but these findings are largely confined to a few compound families and cell types. A handful of studies in APP/PS1 transgenic mice have reported reductions in plaque burden following lignan treatment, though the number of such studies remains limited and the dosing regimens vary considerably across reports. It is also worth noting that many of the lignans with reported anti-Aβ activity in vitro have not been systematically evaluated in vivo, and direct comparisons between different structural types are scarce.

Direct binding to Aβ and subsequent inhibition of aggregation represent one of the principal mechanisms that have been investigated for lignans in the context of AD. At the molecular level, these compounds are thought to interact with specific amino acid residues of Aβ through hydrogen bonds and hydrophobic contacts, thereby disrupting intermolecular hydrophobic associations and β-sheet formation, which, in turn, impedes oligomerization and fibrillization. This mechanistic model, however, is largely derived from molecular docking simulations and needs to be interpreted with caution. Taking a pair of propanol-type lignan enantiomers from *Prunus tomentosa* seeds (compounds 63 and 64) as an example, Thioflavin T (ThT) fluorescence assays showed that both inhibited Aβ aggregation by 63.25 ± 2.68% and 67.13 ± 0.90%, respectively—values that were significantly higher than those of the positive control curcumin in the same assay. Molecular docking work further suggested that their binding region overlaps with that of curcumin, targeting the Gln15 residue of Aβ. While these biochemical data clearly demonstrate anti-aggregation activity in a cell-free system, it should be noted that such in vitro potency does not necessarily predict efficacy in cellular or animal models, let alone in humans [96]. The tetrahydrofuran-type neolignan schibitubin H (11) was evaluated in a series of in vitro assays. In cell-free ThT-based aggregation tests, it exhibited inhibitory activity against Aβ fibril formation, and in SH-SY5Y cell cultures exposed to Aβ_25–35_, it also showed capacity to reduce oxidative stress and hypoxia-related injury. Notably, at a concentration as low as 3.2 nM, it significantly protected neurons from Aβ-induced damage; at 2 µM, the survival rate of Aβ_25–35_-challenged SH-SY5Y cells reached 88.6 ± 1.17%. Based on these cellular observations and structural comparisons, the combination of its tetrahydrofuran ring system and epoxy substituent has been proposed as a likely contributor to its potent anti-aggregation activity. So far, the proposed SAR is largely correlative. Confirmatory studies, such as co-crystallization or mutagenesis, are still required to identify the key structural determinants [72].

Targeting the Aβ metabolic pathway to inhibit its production represents an important approach by which lignans intervene in Aβ pathology. These compounds primarily act by inhibiting β-secretase (BACE1) and even γ-secretase activity, thereby blocking the aberrant cleavage of APP, reducing the generation of toxic fragments such as Aβ_1–40_ and Aβ_1–42_, and curbing Aβ aggregation and deposition at the source. In terms of Aβ production and aggregation, several neolignans from Magnolia officinalis have been investigated. The neolignan 4-O-methylhonokiol (35) has been examined for its effects on the amyloidogenic pathway. In cell-based assays, it was reported to lower BACE1 protein levels and enzymatic activity, and similar observations were made for its analogue obovatol (36) in cortical and hippocampal tissue preparations. These in vitro findings are consistent with the idea that these compounds may reduce Aβ production, although the concentrations used in some studies were relatively high compared to what might be achievable in vivo. In transgenic AD models (Tg2576 and APP/PS1 mice), both compounds were reported to reduce Aβ_1−40_ levels and plaque burden, and to upregulate Aβ-degrading enzymes. Cognitive improvements were also observed in these animals, and no overt toxicity was noted at the doses tested. These results are encouraging, but we should keep a few limitations in mind. First, the pharmacokinetic properties of these compounds, especially their oral bioavailability and brain penetration, have not yet been fully defined. Second, the effective doses in mice were far higher than what a typical diet could provide. Third, the number of animals per group in some studies was relatively small, and the duration of treatment was short relative to the chronic nature of AD. Thus, although these two neolignans represent interesting leads for further investigation, the current evidence is still preliminary. While the BBB permeability and in vivo efficacy of these two compounds are encouraging, a few caveats remain. Their pharmacokinetic profiles have not been fully characterized, and long-term safety data in aged animals are still lacking. Thus, although they represent interesting lead candidates, further preclinical work is needed before clinical translation can be considered [83].

Regulating autophagy and mitochondrial function to accelerate Aβ clearance while alleviating the secondary pathological damage triggered by Aβ aggregation constitutes an important synergistic mechanism by which lignans combat Aβ pathology. Magnolol (50), derived from Magnolia officinalis, activates the AMPK/mTOR/ULK1 signaling pathway, upregulates the expression of autophagy-related proteins such as Beclin-1 and LC3-II, degrades p62/SQSTM1, and promotes autophagosome formation, thereby accelerating intracellular autophagic clearance of Aβ and significantly reducing amyloid plaque deposition in the brains of APP/PS1 mice. Concomitantly, it downregulates the expression of cleaved caspase-9 and Bax, upregulates Bcl-2 levels, and blocks the Aβ-induced mitochondrial apoptotic pathway, thereby achieving synergistic neuroprotection through “autophagy regulation-anti-apoptosis [81]. Honokiol (34), on the other hand, targets mitochondrial function regulation by upregulating the expression and activity of mitochondrial deacetylase sirtuin 3 (SIRT3), activating SIRT3-mediated mitophagy, promoting the clearance of damaged mitochondria, ameliorating Aβ oligomer (AβO)-mediated mitochondrial dysfunction, reducing mitochondrial ROS generation, and stabilizing mitochondrial membrane potential. Furthermore, it enhances ATP production through SIRT3 regulation, improves cerebral energy metabolism, and reverses early memory deficits in PS1V97L transgenic mice. Its effects in promoting Aβ clearance and protecting neurons can be specifically blocked by the SIRT3 inhibitor 3-TYP, confirming that SIRT3 is its core target in combating Aβ pathology. In addition, HKL can inhibit astrocyte A1 polarization by modulating the SIRT3-STAT3 axis, thereby alleviating neuroinflammation triggered by Aβ aggregation and further attenuating the cascade damage of Aβ pathology [139].

The broad-spectrum anti-Aβ activity of lignans with diverse structural subtypes further enriches the structural diversity and mechanistic repertoire of anti-AD natural products. Lignans of different scaffolds complement each other through distinct modes of action, thereby refining the intervention network by which lignans target Aβ pathology. The 7,8-seco-neolignan schibitubin I (79) focuses on regulating Aβ metabolism and improving cellular energy metabolism. It attenuates Aβ_25–35_-induced neuronal toxicity at a concentration as low as 3.2 nM, and at 2 μM, it raises the survival rate of injured cells to 85.1 ± 2.70%. By modulating pathways related to Aβ production and clearance and ameliorating energy supply deficits in the AD brain, this compound exerts anti-Aβ effects, thereby filling a gap in the research on seco-neolignans against AD. The dibenzylbutane-type lignans Schibitubin C (1) and Galgravin possess dual activities of both anti-Aβ aggregation and alleviation of Aβ toxicity. At a concentration as low as 3.2 nM, Schibitubin C significantly increases the survival rate of SH-SY5Y cells injured by Aβ_25–35_, aligning with the multiple pathological features of “Aβ toxicity + oxidative stress + hypoxic injury” in the AD brain. Galgravin (12), by directly inhibiting Aβ aggregation and scavenging reactive oxygen species, increases the survival rate of Aβ-injured model cells to 86.7 ± 1.30% at a concentration of 2 μM, demonstrating potent Aβ pathology-intervening activity. The tetrahydrofuran-type lignan (−)-Nectandrin A (13) and the benzyltetralin-type lignan (−)-Isootobaphenol (4) exert neuroprotection through the synergistic effect of “anti-Aβ aggregation-antioxidant/anti-apoptosis”; the former shows prominent protective activity against H_2_O_2_-induced oxidative damage at 3.2 nM, while the latter blocks Aβ oligomer formation and inhibits the activation of apoptotic signaling pathways. Both achieve effective intervention against Aβ pathology at low concentrations, providing an important basis for structure–activity relationship studies of lignans [72]. In addition, sesamin (39) from sesame can directly target the core pathology of AD by inhibiting abnormal Aβ aggregation. It also possesses anti-inflammatory and antioxidant activities: it downregulates pro-inflammatory cytokines in the brain and alleviates the neuroinflammation triggered by Aβ aggregates [140]. Together, these effects constitute a dual regulatory mechanism—directly countering Aβ while indirectly suppressing Aβ-driven inflammatory damage [141].

**Figure 3:**
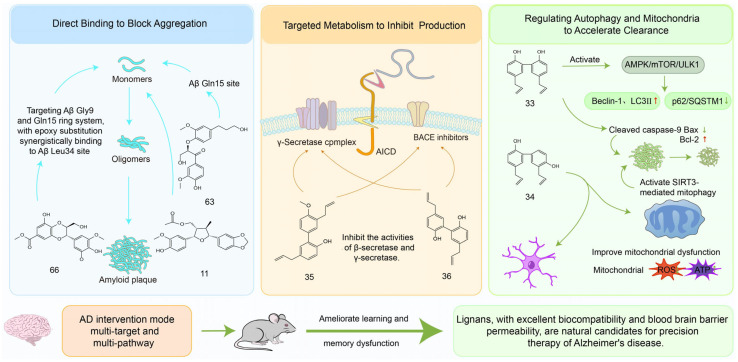
Schematic diagram of the mechanism of lignans for the treatment of Alzheimer’s disease by alleviating Aβ aggregation.

In summary, lignans mediate multi-dimensional targeted intervention against Aβ pathology through three core pathways: directly binding to Aβ targets to block aggregation, inhibiting BACE1/γ-secretase to reduce production, and activating autophagy/mitochondrial pathways to accelerate clearance (Figure 3). Moreover, the majority of these lignans also possess synergistic activities such as antioxidant, anti-inflammatory, anti-apoptotic, and energy metabolism-improving effects, which can effectively block the downstream pathological cascades triggered by Aβ aggregation and significantly ameliorate cognitive dysfunction in AD model animals. Clear structure–activity relationships exist for the anti-Aβ activity of lignans with different skeleton types, where stereochemistry, ring systems, substituent types and positions all exert critical influences on their activity and binding specificity—for example, the stereoselectivity of 8′,9′-epoxy neolignans, the synergistic effect of epoxy substitution in tetrahydrofuran-type lignans, and the essential C-3 trans double bond in benzofuran neolignans—thereby providing an important theoretical basis for the structural optimization, lead compound screening, and druggability modification of lignan compounds. With their favorable biocompatibility, BBB permeability, and multi-target synergistic advantages, lignan compounds have become an important direction in the development of anti-AD natural drugs. Future efforts should further pursue in-depth investigations of their in vivo pharmacokinetics and structure–activity relationships, combine nanoformulation and other delivery systems to improve their bioavailability, and concurrently conduct multicenter clinical studies to verify their clinical efficacy and safety, thereby advancing their translation from preclinical research to clinical treatment of AD and providing novel natural drug candidates for precise, multi-mechanism therapy of Alzheimer’s disease.

### 3.2. Targeting Oxidative Stress

Oxidative stress is a core pathological driver of AD and a key hub connecting aging with the various pathological pathways of AD. It is characterized by excessive accumulation of ROS and impairment of the endogenous antioxidant defense system, which subsequently lead to neuronal oxidative damage, apoptosis, and cognitive dysfunction. Lignans have been shown to exert neuroprotective effects by modulating oxidative stress-related signaling pathways, scavenging free radicals, and enhancing antioxidant enzyme activities. The evidence for these effects comes predominantly from cell culture models (e.g., H_2_O_2_- or SNP-treated PC12 and SH-SY5Y cells), with a smaller number of studies using rodent AD models. While the mechanistic details are well elucidated in these systems, their direct applicability to human AD requires further validation.

The furofuran lignans isolated from the leaves of *Eucommia ulmoides*, including (−)-7-epipinoresinol mr1 (42), (+)-Medioresinol (43), and (+)-dihydropinoresinol (44), exert neuroprotective effects by modulating the key regulatory axis of the cellular antioxidant response—the PI3K/AKT/GSK-3β/Nrf2 signaling pathway. These compounds upregulate the protein expression of heme oxygenase-1 (HO-1), NAD(P)H:quinone oxidoreductase 1 (NQO-1), and catalase (CAT) at both the transcriptional and translational levels while enhancing the enzymatic activities of superoxide dismutase (SOD) and glutathione peroxidase (GPx). By selectively scavenging pathological intracellular ROS and alleviating oxidative stress-induced neuronal dysfunction, these compounds establish a robust antioxidant defense network in neural cells [80]. Other lignans from *Eucommia ulmoides* leaves (37, 49, 50, 51, 77, 78) also exhibited dose-dependent neuroprotective activity in the H_2_O_2_-induced PC12 cell injury model, as evidenced by increased cell viability, reduced lactate dehydrogenase (LDH) release and ROS production, and restored SOD and GPx activities, confirming that Eucommia lignans possess general antioxidant potential against AD-related oxidative damage [84].

Benzofuran neolignans represent an important subclass within the lignan family that possesses potent anti-AD activity, with (+)-licarin A (89), eupomatenoid-7 (90), and eleganal (93) isolated from *Phyllanthus reticulatus* serving as typical representatives. In a sodium nitroprusside (SNP)-induced PC12 cell injury model that mimics AD-related oxidative stress, these three compounds at 10 μM exhibited superior neuroprotective effects compared to the clinical cerebral protective agent edaravone, and displayed favorable concentration dependence within the range of 2.5–10 μM without evident cytotoxicity. Their core mechanism of action involves antagonizing the excessive accumulation of nitric oxide (NO) induced by sodium nitroprusside, inhibiting oxidative stress-mediated lipid peroxidation and neuronal apoptosis, and maintaining neuronal cell survival and functional integrity. This closely aligns with the pathological processes in AD, which are characterized by NO-mediated exacerbation of oxidative stress and progressive neuronal loss. Based on the limited set of benzofuran neolignans tested in this study, some preliminary structure–activity trends can be tentatively inferred. The presence of a trans double bond at C-3 appeared to correlate with higher antioxidant activity in the SNP-induced PC12 assay, and a 4-hydroxy substitution was associated with greater neuroprotective effects than a 5-hydroxy substitution in the same system. Stereochemistry, by contrast, did not seem to have a major impact on the measured activity in this particular assay. However, these observations should be interpreted with caution. The conclusions are drawn from a small number of compounds within a single structural class, and the activity differences were relatively modest. Furthermore, these SAR trends have only been examined in one cell-based assay (SNP-induced oxidative stress in PC12 cells) and have not been validated in other oxidative stress models or in vivo. It is also unclear whether these structural features influence other properties relevant to drug development, such as metabolic stability, solubility, or brain penetration. Thus, while these initial observations may offer some guidance for further compound design, they should not be taken as firmly established SAR principles [137].

Dibenzylbutane-type lignans—characterized by a C8–C8′-linked bis-phenylpropane scaffold—possess broad-spectrum anti-oxidant and neuroprotective activities against AD due to the synergistic substitution of hydroxyl, methoxy, and methylenedioxy groups. Schibitubin C (1), Schibitubin D (2), Galgravin (12), and Oleiferin F (3), isolated from the fruits of *Schisandra tuberculata*, can target the multiple pathological features of “oxidative stress + Aβ toxicity + hypoxic injury” in the AD brain. Schibitubin C (1), at a concentration as low as 3.2 nM, significantly increases the viability of SH-SY5Y cells subjected to hypoxic injury induced by Aβ_25–35_ and cobalt chloride, while its acetylated derivative, Schibitubin D (2), maintains stable antioxidant activity in a hydrogen peroxide-induced oxidative stress model and exhibits markedly improved biocompatibility, providing a typical paradigm for the druggability optimization of lignans. Galgravin (12) exerts potent anti-Aβ toxicity effects by inhibiting Aβ aggregation and alleviating oxidative stress, while Oleiferin F (3) demonstrates comprehensive protective effects against hypoxia, oxidative stress, and Aβ-induced injury, with antioxidant activity comparable to that of vitamin E, thereby validating the stable anti-AD activity pattern of the “dibenzylbutane skeleton + multi-substituent modification” [72]. Dibenzylbutyrolactone-type lignans isolated from *Wikstroemia alternifolia*, including the newly discovered wikstrolignan B (16) and the known pluviatolide (17), honokiolin (18), isocubebin (5), and sanshodiol (6), demonstrate neuroprotective activity efficacy superior to that of edaravone 10 μM in the sodium nitroprusside-induced PC12 cell model. By antagonizing excessive NO accumulation and inhibiting oxidative stress-mediated neuronal apoptosis, these compounds further consolidate the central role of dibenzylbutyrolactone-type lignans among anti-AD natural active ingredients targeting oxidative stress [73].

Specific lignans can regulate oxidative stress by precisely activating antioxidant signaling pathways. (+)-Lariciresinol (67), isolated from the roots of *Rubia philippinensis* (Philippine madder), is a typical representative that targets the p38-Nrf2-ARE pathway. This optically active lignan activates the p38 mitogen-activated protein kinase (MAPK) signaling pathway, and promotes the release of nuclear factor erytroid 2-related factor 2 (Nrf2) from cytoplasmic sequestration by Kelch-like ECH-associated protein 1 (Keap1) and its subsequent nuclear translocation, thereby upregulating the transcriptional and translational levels of downstream antioxidant enzymes (SOD1, GPx1, CAT) and phase II detoxifying enzymes (HO-1, NQO1). Pharmacological inhibition experiments confirmed that the Nrf2 inhibitor brusatol and the p38 inhibitor SB239063 blocked the antioxidant effect of lariciresinol, thereby clarifying the specific molecular mechanism by which this compound establishes a multi-layered cellular antioxidant defense network [97]. Gomisin N (30), isolated from *Schisandra sphenanthera* (commonly known as Zhongjinliu), exerts anti-AD effects by modulating the PI3K/GSK3β/Nrf2 signaling axis: it upregulates the protein expression of phosphorylated glycogen synthase kinase 3β (pGSK3β), Nrf2, and its downstream target genes NQO1 and HO-1 in AD model animals and cells, promotes Nrf2 nuclear translocation and downstream gene transcription, and enhances the body’s antioxidant stress capacity to defend against oxidative damage. The PI3K inhibitor LY294002 can reverse the aforementioned protein expression changes, confirming the core mediating role of this pathway in the antioxidant neuroprotective effect of Gomisin N [142]. Furthermore, Sauchinone (93) treatment improves the phosphorylation level of Nrf2 and reduces its interaction with Keap1. Through the multi-step synergy of inhibiting GSK3 phosphorylation, activating PKC, and upregulating Nrf2 activity, it strengthens the Nrf2-mediated anti-oxidant defense system. Sauchinone markedly reduces hepatic oxidative stress in models of acetaminophen-induced liver injury and high-fat diet-induced steatosis. These findings offer compelling translational evidence that sauchinone could similarly mitigate neuronal oxidative damage and protect against neurodegeneration in AD [138]. The furofuran-type lignan glucosides Aurantioside C (54) and Aurantioside D (55), isolated from the leaves of *Osmanthus fragrans var. aurantiacus*, provide structurally novel and mechanistically defined natural candidate molecules for AD treatment. Both feature a furofuran lignan core skeleton, with various phenylpropanoyl substituents such as feruloyl and sinapoyl groups attached via glucosidic bonds. The abundant methoxy substitutions in their structures serve as an important structural basis for their activity. In an L-glutamate-induced HT22 hippocampal neuronal injury model, Aurantioside D (55) exhibited the most prominent activity, with a neuroprotection rate of 88.3% at 50 μM (EC_50_ = 15.5 ± 3.5 μM), significantly outperforming the positive control Trolox. Aurantioside C (54) showed the next best activity, with a protection rate of 66.5% at 50 μM (EC_50_ = 37.4 ± 1.2 μM). The mechanism by which both compounds target oxidative stress, a core pathological process in AD, is clearly defined: they form stable hydrogen bonds and hydrophobic interactions with key amino acid residues such as Ser363, Asn414, and Arg415 of the Keap1 protein, thereby disrupting the formation of the Keap1/Nrf2 complex, promoting Nrf2 nuclear translocation, and activating downstream ARE-mediated expression of antioxidant enzymes such as HO-1, ultimately alleviating neuronal oxidative damage; Western blot analysis further confirmed that Aurantioside D (55), at a concentration of 10 μM, significantly downregulated the glutamate-induced overexpression of Keap1 protein and restored cellular redox homeostasis [92]. The novel norlignans Capitugenin A (83) and Capitugenin C (84), isolated from *Tupistra wattii*, exhibited concentration-dependent neuroprotective effects in a glutamate-induced SH-SY5Y cell oxidative injury model, with Capitugenin C (84) demonstrating superior antioxidant activity compared to N-acetylcysteine (NAC). Capitugenin C (84) activates the Nrf2/HO-1 antioxidant signaling axis, strengthens the endogenous cellular antioxidant defense system, significantly increases SOD activity, reduces LDH release and ROS accumulation, and simultaneously promotes the expression of brain-derived neurotrophic factor (BDNF), thereby exerting dual pharmacological effects of anti-oxidative damage and neurotrophic action. This reveals a novel mechanism by which norlignans intervene in the pathological process of AD through the Nrf2/HO-1-BDNF pathway [132].

The neolignans 4-O-methylhonokiol (35) and honokiol ether (36), isolated from *Magnolia officinalis*, by virtue of their unique phenolic ring structures and favorable blood–brain barrier permeability, exert multi-target synergistic regulatory effects against oxidative stress in AD. 4-O-methylhonokiol can restore glutathione levels in the brain, inactivate p38 MAP kinase to reduce ROS production, inhibit protein carbonylation and the accumulation of lipid peroxidation products, and significantly alleviate oxidative damage in PS2 mutant mice and Aβ-induced models. Simultaneously, 4-O-methylhonokiol blocks the neuronal apoptotic pathway by inactivating apoptosis-related proteins such as BAX and caspase-3, and reverses learning and memory deficits in AD transgenic mice by ameliorating the oxidative stress and inflammatory microenvironment, with a favorable safety profile at therapeutic doses. Honokiol ether can regulate brain oxidative stress levels, and synergistically enhance neuroprotective effects, and short-term intervention at doses of 0.2–1 mg/kg/d significantly improves cognitive function in AD model animals, demonstrating promising application prospects in alleviating oxidative-stress-related cognitive impairment [83].

Additional lignans and their derivatives also exhibit robust anti-AD antioxidant neuroprotective effects through multiple mechanisms. HKL (34) upregulates the expression and activity of mitochondrial deacetylase sirtuin 3 (SIRT3), promotes adenosine triphosphate (ATP) production, inhibits mitochondrial ROS generation, improves mitochondrial energy metabolism and alleviates oxidative stress, reverses mitochondrial dysfunction mediated by Aβ oligomers, and rescues memory deficits in PS1V97L transgenic mice; its antioxidant effect can be blocked by the SIRT3 inhibitor 3-TYP [139]. Schisandrin A (28) increases the activity of glutathione peroxidase (GSH-Px) and the content of glutathione (GSH) in the cerebral cortex and hippocampus of AD model rats, reduces the levels of malondialdehyde (MDA) and oxidized glutathione (GSSG), and ameliorates cognitive dysfunction through antioxidative stress pathways [78]. Schisandrin A (31) increases the levels of SOD and GSH in neuronal cells, reduces the content of MDA and ROS, scavenges hydrogen peroxide, organic peroxides, and oxygen free radicals by regulating GSH-dependent biochemical parameters, blocks the lipid peroxidation chain reaction, and protects brain tissue from oxidative stress damage [79,143]. Schisandrin B (32) suppresses ROS generation and inhibits nicotinamide adenine dinucleotide phosphate (NADPH) oxidase in microglial cells, thereby alleviating neuroinflammatory damage mediated by oxidative stress. The glycosylated lignan derivatives 85 and 86, isolated from the flower buds of *Magnolia biondii*, exhibited significant neuroprotective activity in a 6-hydroxydopamine-induced SH-SY5Y cell injury model, with half-maximal inhibitory concentration (IC_50_) values both lower than that of curcumin, revealing the potential value of glycosylated lignans in antagonizing neuronal oxidative damage and inhibiting neuronal apoptosis [80]. Lyciumamide A (87), a phenolic amide dimer with lignan-like antioxidant activity, can reverse N-methyl-D-aspartate (NMDA)-induced intracellular calcium overload and excessive ROS production, inhibit the activation of oxidative stress-related signaling molecules such as p-NR2B, p-CaMKII, p-JNK, and p-p38, and exert brain injury protection by blocking NMDA receptors and suppressing mitochondrial oxidative stress and apoptosis [134].

**Figure 4:**
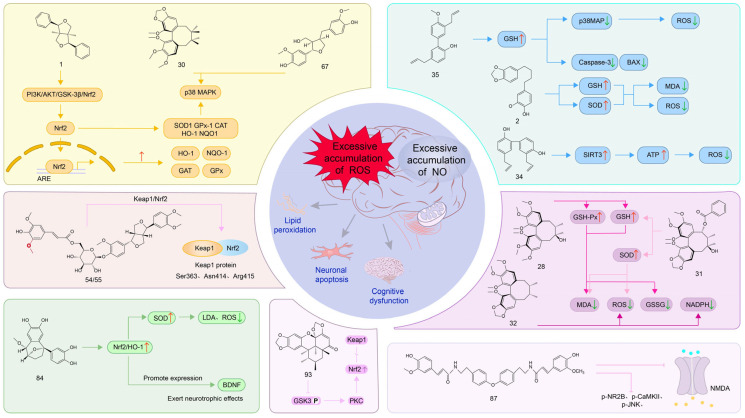
Schematic diagram of the mechanism of lignans in treating Alzheimer’s disease by alleviating oxidative stress. 1 is a dioxolane-type lignan isolated from *Eucommia ulmoides* leaves, and 2 is a dibenzylbutane-type lignan.

Overall, lignans derived from a variety of medicinal plants act as multi-target geroprotective agents, counteracting oxidative stress in Alzheimer’s disease through a synergistic mechanistic network. They modulate core antioxidant signaling axes, including PI3K/AKT/GSK-3β/Nrf2, p38-Nrf2-ARE, and SIRT3; scavenge excessive ROS and NO radicals; enhance the expression and activity of endogenous anti-oxidant enzymes; improve mitochondrial energy metabolism; and inhibit oxidative-stress-mediated neuronal apoptosis and lipid peroxidation (Figure 4). Structure–activity relationship (SAR) studies have identified several pharmacophoric features critical for anti-AD activity. For instance, the C-3 trans double bond in benzofuran neolignans plays a key role, and multiple substituents on the dibenzylbutane scaffold act in a synergistic manner. These insights, combined with prodrug optimization strategies such as acetylation, glycosylation, and other structural modifications, lay a practical foundation for designing lignan-based drug candidates against Alzheimer’s disease. Most lignans have demonstrated favorable safety and efficacy in preclinical Alzheimer’s disease models, and their multi-target antioxidant properties align perfectly with the highly interconnected, aging-driven pathological features of AD. Future in-depth research focused on pharmacokinetic optimization, precise target identification, ferroptosis–oxidation crosstalk, gut–brain axis regulation, and multi-center clinical trials will accelerate the translation of lignans from the laboratory to the clinic, opening new avenues for natural product-based, redox-targeting interventions against age-related neurodegenerative diseases.

### 3.3. Targeting Neuroinflammation

Neuroinflammation, as a key link in the core pathological process of AD, is primarily characterized by excessive microglial activation, massive release of pro-inflammatory cytokines, and aberrant activation of downstream inflammatory signaling pathways. Persistent central neuroinflammation can accelerate Aβ deposition, tau hyperphosphorylation, and neuronal apoptosis, ultimately exacerbating cognitive dysfunction. A wealth of preclinical data support the anti-neuroinflammatory efficacy of lignans, with most functional verification conducted on LPS-activated BV2 and N9 microglial cell lines. Comparatively few studies have further validated these protective effects in rodent models simulating pathological neuroinflammation and Alzheimer’s disease progression. Accumulated mechanistic research at the cellular and animal levels repeatedly identifies TLR4/NF-κB, MAPK and NLRP3 inflammasome as the core signaling pathways modulated by lignans. Nevertheless, the majority of available experimental evidence originates from short-term acute LPS or NMDA intervention studies, which cannot fully recapitulate the long-term pathological progression of clinical Alzheimer’s disease. At present, there is still a complete lack of clinical trial data to support the clinical translational application of lignans against AD.

Dibenzocyclooctadiene-type lignans are the core active constituents of Schisandra plants, and their regulatory effects on AD-related neuroinflammation have been fully validated by in vitro and in vivo experiments. A series of eleven dibenzocyclooctadiene-type lignans (19–28) from *Schisandra chinensis* fruits were tested for their ability to inhibit LPS-induced NO production in BV2 microglial cells, and all showed some degree of inhibition This variation suggests that even within the same structural class, minor differences in substituent patterns can have a major impact on activity [78]. For gomisin A (29), further mechanistic studies in N9 microglial cells revealed concentration-dependent suppression of iNOS and COX-2 expression, as well as reduced production of NO, prostaglandin E_2_(PGE_2_), TNF-α, IL-1β, and IL-6. While these cellular data are relatively consistent across multiple inflammatory markers, it should be noted that the concentrations required for these effects are considerably higher than those that would be expected to be achieved in the brain following oral administration. Moreover, the relevance of LPS-stimulated microglial cell lines to the chronic, low-grade neuroinflammation seen in AD is uncertain. Thus, while this body of work provides useful insights into the anti-inflammatory potential of dibenzocyclooctadiene lignans in vitro, their in vivo relevance remains to be established. Its anti-inflammatory mechanism primarily relies on blockade of the TLR4–NF-κB–MAPKs signaling axis by downregulating TLR4 protein expression and inhibiting NF-κB nuclear translocation and MAPKs pathway phosphorylation while simultaneously reversing LPS-induced ROS generation and the upregulation of NADPH oxidase activity, thereby alleviating microglia-mediated apoptosis of SH-SY5Y cells, primary cortical neurons, and hippocampal neurons [144]. Furthermore, Schisandrin A (31), derived from *Schisandra chinensis*, can attenuate Aβ-induced inflammatory injury by downregulating the expression of pro-inflammatory cytokines such as IL-6, IL-1β, and TNF-α, an effect associated with the modulation of the ERK/MAPK signaling pathway [79,145]. Schisandrin B (32) targets the TLR4-dependent MyD88/IKK/NF-κB signaling pathway, inhibiting the interaction of TLR4 with the adaptor proteins MyD88, IRAK-1, and TRAF-6, thereby blocking NF-κB pathway activation. It simultaneously reduces microglial ROS production and NADPH oxidase activity, significantly downregulates the release of inflammatory mediators such as NO, TNF-α, and PGE_2_, and exerts a marked protective effect against inflammatory neuronal injury in microglia–neuron coculture systems [80,146].

The dihydrofurofuran-type lignans include the aschantin epimers AM2 (52) and AM3 (aschantin, 53). Both compounds were non-cytotoxic at 5–20 μM, as confirmed by MTT and LDH assays, and they potently inhibited LPS-induced activation of BV2 microglia. Mechanistically, they downregulated the protein expression of iNOS and COX-2 in a concentration-dependent manner; at 20 μM, their inhibition of iNOS even exceeded that of the classical anti-inflammatory drug dexamethasone. These effects led to a marked reduction in excessive NO and PGE_2_ production, along with decreased release of pro-inflammatory mediators such as IL-6, TNF-α, and monocyte chemoattractant protein-1 (MCP-1). Their core anti-inflammatory mechanism involves dual blockade of the classical MAPK/NF-κB inflammatory signaling pathway by inhibiting the phosphorylation of ERK, JNK, and p38, downregulating the expression of phosphorylated inhibitor of κB α (p-IκBα) and phosphorylated p65 (p-p65), and blocking the nuclear translocation of the p65 subunit, thereby suppressing the expression of pro-inflammatory genes at the transcriptional level. Notably, the configurational difference at the H-7 position between the two compounds did not significantly affect their anti-inflammatory activity or mechanism [91]. This provides an important structure–activity relationship basis for the structural modification of this class of lignans.

Benzylbutyrolactone-type and neolignan compounds exert a synergistic effect of inflammatory intervention and neuroprotection via multi-target modulation. The dibenzylbutyrolactone-type lignans from *Wikstroemia alternifolia*—Wikstral ternifol B (16), Pluviatolide (17), Hinokinin (18), Isocubebin (5), and Sanshodiol (6)—antagonize excessive NO accumulation induced by sodium nitroprusside (SNP) and inhibit oxidative stress-mediated neuronal apoptosis. Their neuroprotective activity at 10 μM surpasses that of the clinical cerebral protective agent edaravone, providing novel natural candidates for AD treatment targeting the oxidative stress–inflammation crosstalk [73]. 2-(((1*R*,2*R*)-1-hydroxy-1-(4-hydroxy-3,5-dime-thoxyphenyl)propan-2-yl)oxy)-3-methoxy-5-((E)-prop-1-en-1-yl)phenol (80), 1-((2*S*,3*S*)-7-hydroxy-2-(4-hydroxy-3-methoxy-phenyl)-3-methyl-2,3-dihydrobenzofuran-4-yl) ethanone (81), and Callislignan B (82), isolated from the roots of *Adelostemma gracillimum*, can protect primary cortical neurons against NMDA receptor-mediated excitotoxicity. Since excitotoxicity can indirectly exacerbate neuroinflammation through microglial activation, these compounds achieve the dual effects of inflammatory regulation and neuroprotection [131]. Savinin (15), derived from *Acanthopanax giraldii* (Huangmaowujia), exerts anti-inflammatory effects through the dual regulation of the MAPK/NF-κB pathway and NLRP3 inflammasome activation. It not only dose-dependently inhibits the phosphorylation of p38, JNK, and ERK and the nuclear translocation of NF-κB p65, downregulating the IKK/IκBα signaling cascade, but also reduces the expression of core inflammasome components such as NLRP3, caspase-1, and ASC, thereby decreasing the production of inflammatory mediators like NO and PGE_2_, as well as that of the enzymes iNOS and COX-2, ultimately alleviating inflammatory pathological damage in hippocampal neurons. Molecular docking confirmed that it can bind to the active sites of MAPK13, NF-κB, and NLRP3 through hydrogen bonds and hydrophobic interactions [77]. This provides a molecular basis for its targeted regulation.

Lignanamides, a special lignan subtype containing amide functional groups, have opened a new direction for AD treatment through their anti-inflammatory activity. All eight lignanamides (68–75) isolated from *Piper hancei Maxim* inhibit LPS-induced NO production in BV-2 microglial cells [102]. Cannabisin (76) concentration-dependently inhibits the secretion and mRNA expression of pro-inflammatory cytokines such as IL-6 and TNF-α, reduces the phosphorylation level of NF-κB p65, and suppresses its nuclear translocation while also attenuating the expression of TLR4 and MyD88, thereby blocking microglia-mediated neuroinflammation through modulation of the TLR4/MyD88/NF-κB pathway [147]. Lyciumamide A (87), a phenolic amide dimer isolated from the fruit of *Lycium barbarum*, can reverse the NMDA-induced increase in ROS generation, inhibit the elevation in p-JNK and p-p38 levels, reduce inflammation-associated neuronal apoptosis by suppressing mitochondrial oxidative stress, and achieve synergistic regulation of anti-inflammatory and antioxidant effects [134].

Lignans from other medicinal plants also modulate neuroinflammation. Sesamin (39) and (−)-sesamin (40), isolated from *Asarum* roots, can downregulate the levels of pro-inflammatory factors such as NF-κB, IL-1β, TNF-α, and IL-6 in the cerebral cortex and hippocampus while modulating the expression of anti-inflammatory factors like IL-10, thereby ameliorating microglial dysfunction induced by high glucose or advanced glycation end products (AGEs) and delaying the progression of central neuroinflammation [87]. Phillyrin (47), through modulating the peroxisome proliferator-activated receptor γ (PPARγ) signaling pathway, inhibits microglial overactivation, and reduces NF-κB phosphorylation and the activation of downstream inflammatory pathways, thereby suppressing the pro-inflammatory responses mediated by activated microglia [89]. Neolignan compounds from *Eucommia ulmoides* leaves, such as Cinchonain Ib (77) and threo-guaiacylglycerol-8-O-4′-sinapyl alcohol ether (78), can reduce ROS levels and enhance the activities of the antioxidant enzymes SOD and GPx, thereby alleviating oxidative stress-induced neuronal damage and indirectly inhibiting microglial activation triggered by oxidative stress, achieving synergistic intervention against both oxidative stress and neuroinflammation [84].

**Figure 5:**
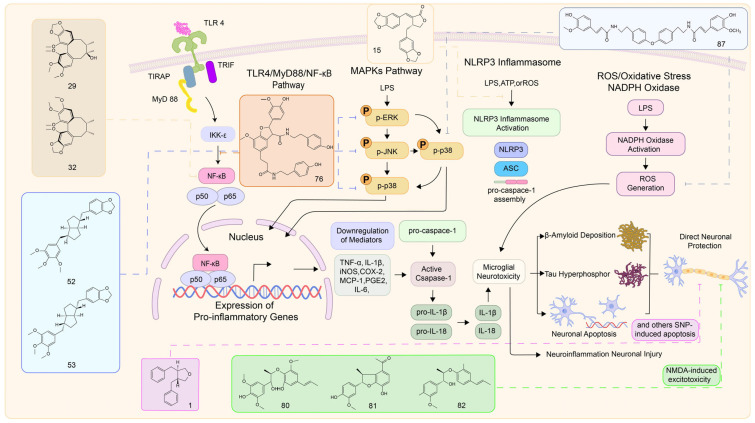
Schematic diagram of the mechanism of lignans against Alzheimer’s disease by inhibiting neuroinflammation.

In summary, lignan compounds can target core aging-associated neuroinflammatory pathways such as TLR4/NF-κB, MAPK, and the NLRP3 inflammasome, inhibit aberrant microglial activation, downregulate the expression of key inflammatory enzymes including iNOS and COX-2, as well as that of pro-inflammatory cytokines such as TNF-α, IL-1β, and IL-6, and concurrently ameliorate key AD pathological processes such as oxidative stress and excitotoxicity, thereby achieving multi-dimensional, multi-target, and network-based intervention against neuroinflammation (Figure 5). Lignans with different skeleton types exhibit clear structural selectivity and pathway cooperativity in inflammatory regulation, greatly expanding the research and development strategies for anti-AD natural products. Moreover, their favorable biocompatibility, blood–brain barrier permeability, and low toxicity provide a robust foundation for subsequent structural optimization, formulation improvement, and clinical translation. Future efforts should further elucidate the structure–activity relationships of the anti-inflammatory effects of lignans, their in vivo pharmacokinetic characteristics, and the regulatory mechanisms of the gut microbiota–gut–brain axis, and conduct high-quality multi-center clinical trials to determine safe and effective doses, thereby advancing lignans toward becoming novel geroprotective natural drugs or clinical adjuvant therapeutic agents targeting neuroinflammation in AD.

### 3.4. Targeting the Cholinergic System

**Figure 6:**
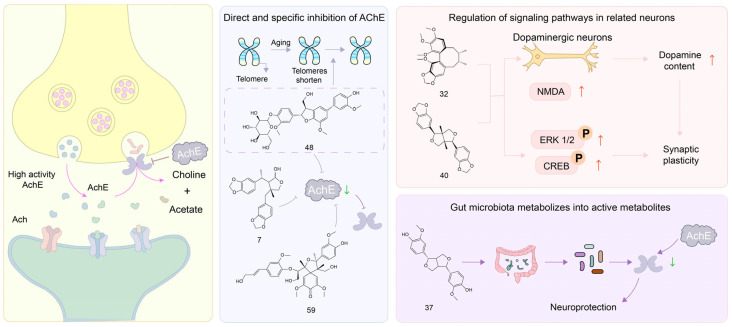
Schematic diagram of the mechanism of lignans in the treatment of Alzheimer’s disease by repairing the cholinergic system.

Cholinergic deficit is a hallmark pathological feature of AD, characterized primarily by degeneration of cholinergic neurons in the basal forebrain, reduced synthesis and release of ACh in the brain, and elevated acetylcholinesterase (AChE) activity, which accelerates ACh degradation in in the synaptic cleft. These changes directly lead to impaired cholinergic neurotransmission and are closely associated with the progressive decline in learning, memory, and cognitive function observed in AD patients. Lignans can exert significant modulatory effects on the cholinergic system through multiple mechanisms, including directly inhibiting AChE activity, maintaining ACh homeostasis in the brain, and regulating cholinergic signal transduction pathways (Figure 6). By virtue of their advantages such as multi-target effects, low toxicity, and favorable biocompatibility, these compounds have emerged as highly promising natural candidates for ameliorating cholinergic deficits in AD, and their pharmacological mechanisms targeting the cholinergic system have been fully validated in in vitro enzyme assays, cellular models, and in vivo AD animal models.

Schisandrin B (32) exerts protective effects against scopolamine-induced neuronal injury by directly modulating core components of the cholinergic system. Its neuroprotective mechanism is closely linked to inhibition of AChE activity and enhancement in cholinergic signaling while concurrently attenuating oxidative stress damage—notably, oxidative stress and cholinergic deficits act synergistically in the pathogenesis of AD. In in vivo animal studies assessed by the passive avoidance task (PAT) and Morris water maze (MWM), scopolamine treatment resulted in markedly impaired learning and memory abilities in rats, accompanied by elevated AChE activity and a sharp decline in endogenous acetylcholine levels in the brain. Pretreatment with Schisandrin B (32) effectively reversed the scopolamine-induced upregulation of AChE activity, and stably maintained the physiological concentration of ACh in the brain, thereby restoring cholinergic neurotransmission function and ultimately ameliorating cognitive impairment caused by cholinergic system dysfunction. This study confirmed the specific regulatory effect of Schisandrin B (32) on the cholinergic system and its potential value in ameliorating AD-related cognitive impairment mediated by cholinergic deficits [148].

Tetrahydrofuran-type lignans derived from *Isatis indigotica* possess dual activities of both neuroprotection and cholinergic system modulation. The nine tetrahydrofuran-type lignans isolated from this plant include three new compounds (58, 59, 62) and two known analogues (60, 61). In vitro activity screening demonstrated that compounds 58,59,61 and 60 conferred neuroprotective effects comparable to those of the positive control Trolox. Target validation further revealed that compound 59 exhibits potent AChE inhibitory activity, thereby directly suppressing AChE-mediated acetylcholine degradation in the brain and ameliorating cholinergic neurotransmission deficits; abnormally elevated AChE activity is one of the core pathological features of AD. The dual activity of this compound makes it an important candidate molecule for intervening in both cholinergic deficits and neuronal injury in AD, demonstrating substantial developmental potential in synergistic therapy targeting multiple pathological processes [149].

Cubebin (7), a classic lignan compound, has had its anti-AD effects and mechanisms targeting cholinergic deficits validated by both in vitro and in vivo experiments. In vitro enzyme assays confirmed that cubebin (7) exhibits significant inhibitory activity against acetylcholinesterase, directly inhibiting the enzymatic degradation of acetylcholine. In scopolamine-induced AD model mice, the animals exhibited significant learning and memory impairment in vivo experiments, accompanied by abnormally elevated AChE activity and enhanced oxidative stress levels in the brain—two pathological processes that interact with and exacerbate each other in AD. Pretreatment with cubebin (7) dose-dependently reversed these pathological alterations: it inhibited the aberrant activation of acetylcholinesterase, restored acetylcholine homeostasis in the brain, and improved cholinergic neurotransmission while also attenuating oxidative stress-induced damage in the brain and reducing the extent of oxidative injury to cholinergic neurons. This dual pharmacological mechanism of “AChE inhibition + oxidative stress alleviation” enables cubebin (7) to effectively ameliorate AD-related cognitive deficits, possessing great potential for further development as an anti-AD drug that exerts synergistic effects against multiple pathological processes [94].

Anisacanthin (48) from *Anisacanthus virgularis* demonstrates potent AChE inhibitory activity in enzymatic assays (IC_50_ of 85 ± 4 nM) and activated telomerase in human melanocytes. However, this compound has not yet been tested in animal models of AD, and its clinical relevance remains entirely speculative at this stage. In addition to its AChE inhibitory effect, Anisacanthin (48) also possesses significant telomerase-activating activity, increasing telomerase activity by 1.64-fold compared to the basal level. Telomerase activation can delay the cellular aging process of neurons, which is consistent with the anti-aging mechanism of action of known telomerase activators such as curcumin. Neuronal aging, in turn, is an important factor accelerating cholinergic neuron degeneration in AD. The synergistic dual activity of “AChE inhibition (targeting cholinergic deficits) + telomerase activation (delaying neuronal aging)” enables Anisacanthin (48) to exert comprehensive modulatory effects on the pathological process of AD, holds immense potential for application in neuroprotection and anti-aging, and shows promise as a multi-target synergistic candidate compound for AD treatment [90].

The major lignan constituent (−)-sesamin (40), isolated from the roots of *Asarum*, does not act through direct inhibition of AChE; instead, it ameliorates AD-related learning and memory impairment by modulating cholinergic and related neuronal signal transduction pathways. In a chronic electric foot shock (EF) stress-induced cognitive impairment model, chronic stress leads to a significant decrease in the phosphorylation levels of extracellular signal-regulated kinase (ERK1/2) and cAMP response element-binding protein (CREB)—two key signaling molecules that regulate neuronal plasticity, learning, and memory. These pathological changes are accompanied by significantly shortened retention latency in the passive avoidance test and a decrease in dopamine content in the nigrostriatal pathway, which indirectly impairs cholinergic neurotransmission by disrupting the balance of neurotransmitter systems. (−)-Sesamin (40) intervention effectively restores the normal phosphorylation levels of ERK1/2 and CREB, elevates dopamine content in the nigrostriatal pathway, and thereby ameliorates the spatial learning and memory and habit memory deficits induced by chronic electric foot shock stress. Its potential mechanism of action is related to the modulation of the N-methyl-D-aspartate (NMDA) receptor and the dopaminergic neuron system, thereby regulating the synaptic plasticity of cholinergic neurons and enhancing the efficiency of cholinergic signal transmission. This study reveals a novel mechanism by which (−)-sesamin (40) improves cognitive function through modulating cholinergic system-related neuronal signaling pathways, and suggests that sesame, a medicinal and edible plant rich in sesamin, holds great potential as a natural resource for the prevention and treatment of AD [87].

Taken together, natural lignans from various plants influence the cholinergic system in AD through multiple mechanisms. One key action is the direct and selective inhibition of acetylcholinesterase (AChE), which reduces acetylcholine breakdown in the synaptic cleft—exemplified by cubebin (7), compound 21 from *Isatis indigotica*, and compound 36. A second mechanism involves indirect modulation of cholinergic signaling via neuronal pathways, thereby enhancing cholinergic function; schisandrin B (32) and (−)-sesamin (40) are representative examples. A third route relies on gut microbiota biotransformation, where parent lignans are converted into active metabolites that exert targeted AChE inhibition and neuroprotection, as seen with bisepoxylignans such as pinoresinol (37).

Moreover, certain lignans can combine cholinergic system modulation with other anti-AD activities, such as alleviating oxidative stress, activating telomerase, and delaying neuronal senescence, thereby exerting synergistic anti-AD effects. This aligns closely with the characteristic complexity and intertwined pathological processes of AD. The structural diversity of lignans results in distinct binding modes and affinities for acetylcholinesterase, and the exploration of their structure–activity relationships provides an important theoretical basis for the structural modification and optimization of lignan-based acetylcholinesterase inhibitors. Most lignans targeting cholinergic deficits have demonstrated good safety and efficacy in preclinical AD models. Their unique advantages, including their medicinal and edible nature and their metabolism mediated by the gut microbiota, offer new insights for the development of novel anti-AD drugs and health products. Future in-depth studies on the pharmacokinetic profiles, target-binding specificity, and multicenter clinical trials of lignans targeting cholinergic deficits will lay a solid foundation for their translation from preclinical research to clinical application, and will also open up new research directions for natural drug therapies for AD based on cholinergic system modulation.

### 3.5. Targeting Neuronal Ferroptosis

Ferroptosis, as an iron-dependent, lipid peroxidation-driven form of programmed cell death, has emerged in recent years as a core novel pathological mechanism in the fields of aging and neurodegeneration. During aging and the progression of AD, brain iron overload, disturbances in glutathione metabolism, decreased GPX4 activity, and the massive accumulation of lipid reactive oxygen species collectively trigger neuronal ferroptosis, directly leading to synaptic loss and irreversible neuronal damage. This process also forms a mutually amplifying vicious cascade with Aβ deposition, Tau hyperphosphorylation, and chronic neuroinflammation, thereby serving as a key downstream node driving AD progression. Targeting the inhibition of ferroptosis has thus become an important emerging strategy for blocking aging-driven neurodegeneration and preserving neuronal survival.

**Figure 7:**
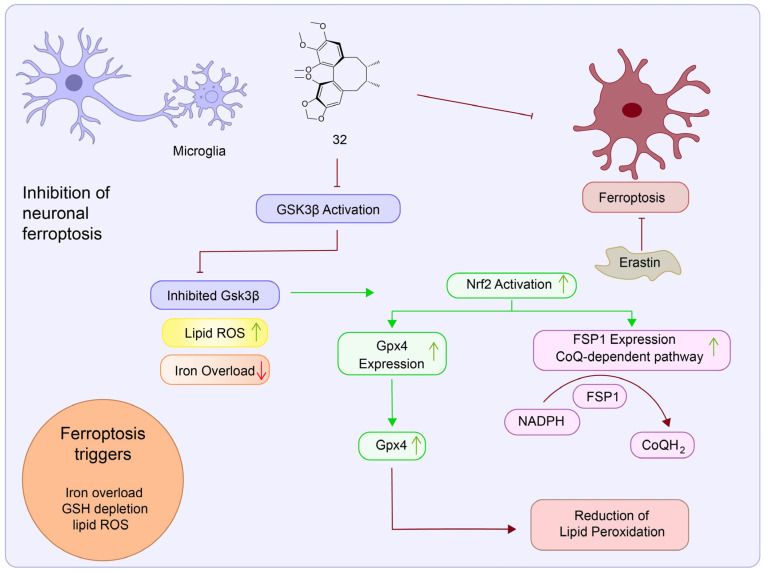
Schematic diagram of the mechanism by which lignans treat Alzheimer’s disease via the ferroptosis pathway.

Schisandrin B (32) has been investigated in this context using erastin-treated SH-SY5Y-695swe cells and microglia–neuron co-cultures. It was found to inhibit glycogen synthase kinase 3β (Gsk3β) and activate Nrf2/GPX4 signaling, reducing ferroptosis and suppressing TNF-α release from ferroptotic neurons. These data are derived entirely from in vitro cell models; in vivo evidence in AD transgenic mice is not yet available. In vitro experiments have confirmed that it can significantly alleviate erastin-induced ferroptosis in SH-SY5Y695swe cells while also regulating the expression of ferroptosis suppressor protein 1 (FSP1), which acts synergistically with the Gsk3β/Nrf2/Gpx4 pathway to enhance the inhibitory effect on ferroptosis. Furthermore, SCH B can block the activation of M1 microglia by inhibiting the release of TNF-α from ferroptotic neurons, thereby achieving multi-pathway regulation of neuroinflammation and ultimately exerting a comprehensive neuroprotective effect (Figure 7) [146].

### 3.6. Targeting the Gut–Brain Axis

**Figure 8:**
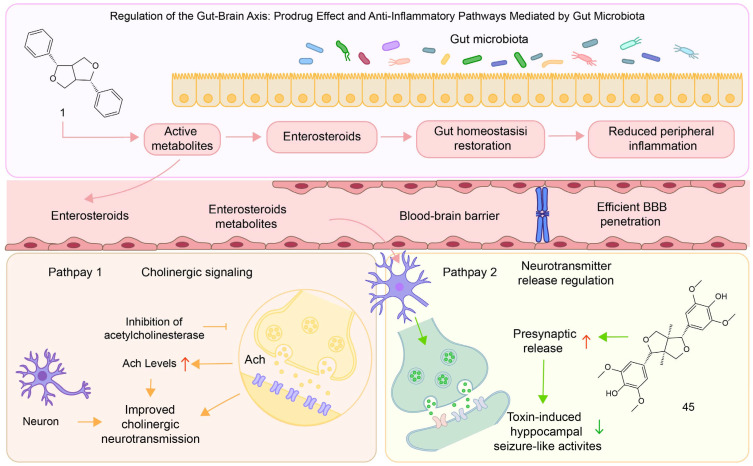
Mechanism diagram of lignans for treating Alzheimer’s disease via regulating gut microbiota. 1 is bicyclic lignans.

The gut microbiota, as a core regulatory component of the gut–brain axis, plays a key driving role in the occurrence and progression of aging-related neurodegenerative diseases. The aging process is accompanied by gut microbiota dysbiosis and increased intestinal barrier permeability, which facilitate the entry of peripheral inflammatory factors, toxic metabolites, and pathogen-associated molecular patterns into the circulatory system. Via neural, immune, and endocrine signaling axes, these factors induce and exacerbate central neuroinflammation, β-amyloid deposition, and Tau hyperphosphorylation, thereby driving the continuous deterioration of the pathological network in AD. In recent years, increasing evidence has indicated that targeting the restoration of gut microbiota homeostasis can effectively block the transmission of peripheral inflammation to the central nervous system, making it an important peripheral target for intervening in aging-driven AD (Figure 8).

Bisepoxylignan compounds exert their anti-AD effects via gut-microbiota-mediated biotransformation into bioactive mammalian lignan metabolites. Representative constituents of this class include syringaresinol (46), pinoresinol (37), sesamolin (38), and pinoresinol monomethyl ether (41). Due to their intrinsic structural characteristics, these compounds cannot exert direct activity in the body and must be m87, olized by the gut microbiota into enterolactone-type metabolites to become effective. The anti-AD effects of enterolactone are mainly manifested in two aspects: first, it acts as a natural AChE inhibitor, reducing the degradation of ACh in the synaptic cleft, maintaining the homeostasis of cholinergic neurotransmitters in the brain, and alleviating memory decline in AD patients; second, through the unique pathway of “gut microbiota metabolism-blood–brain barrier penetration-targeted neuroprotection,” it exerts targeted protective effects on damaged neurons, providing a novel strategy for gut microecological modulation-assisted therapy in AD [85]. Furthermore, its structural analogue (+)-syringaresinol (45) can modulate neurotransmitter release, promote presynaptic transmitter release, and protect against toxin-induced epileptiform activity in hippocampal slices by inhibiting indomethacin (INDO), indirectly corroborating the potential value of bisepoxylignans in the regulation of neuronal signal transmission [88].

### 3.7. Other Novel Anti-AD Mechanisms

**Figure 9:**
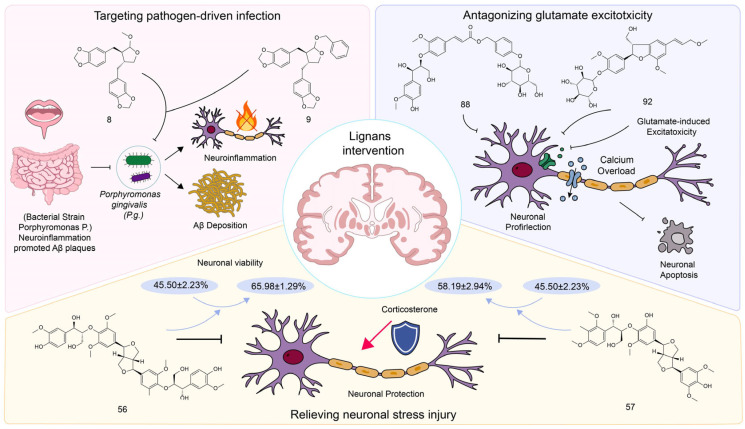
Schematic diagram of the mechanism of lignans in the treatment of Alzheimer’s disease via alternative pathways.

Beyond the core mechanisms outlined above, lignans can also exert neuroprotective effects through novel mechanisms such as inhibiting pathogenic microbial infection, antagonizing glutamate excitotoxicity, and alleviating neuronal stress-induced injury, further enriching their multi-target, network-based anti-AD effect system (Figure 9).

#### 3.7.1. Targeting Pathogenic Microbial Infection

Certain lignans can intervene in infection-associated AD by inhibiting pathogenic bacterial strains, offering a novel interventional strategy for mitigating a significant, yet not fully established, environmental risk factor for AD. Using (−)-cubebin (7) from *Piper cubeba L. f*. as a lead compound, the structurally modified derivatives (−)-O-methyl cubebin (8) and (−)-Obenzyl cubebin (9) can significantly inhibit the growth of Porphyromonas gingivalis. As a well-established AD-associated pathogen, this bacterium can accelerate disease progression by activating neuroinflammation and promoting Aβ deposition [74]. These lignan derivatives, by targeting this pathogenic bacterium, establish a novel “lignan-antibacterial-neuroprotective” action pathway that could potentially help break the cycle of infection-driven peripheral inflammation that contributes to the worsening of central Aβ/tau pathology.

#### 3.7.2. Targeting Glutamate Excitotoxicity

Certain lignan glycosides can effectively antagonize glutamate-induced neuronal excitotoxicity, demonstrating outstanding potential for neuronal protection in the early stages of AD. The neolignan glycosides Bletineoside C (87) and Bletineoside D (88), isolated from the pseudobulbs of *Bletilla striata*, can significantly enhance the survival rate of glutamate-injured PC12 cells, with an efficacy comparable to that of the clinical drug memantine. By inhibiting excitatory amino acid toxicity and reducing calcium overload and neuronal apoptosis, these compounds further expand the application scope of lignan glycosides in neurodegenerative diseases, offering novel structural scaffolds for early neuroprotection in AD [136].

#### 3.7.3. Targeting Neuronal Stress Injury

The lignan compounds first isolated from salted *Aconiti lateralis Radix Praeparata* are important natural candidate molecules targeting neuronal stress-induced injury in the AD brain, including hedyotisol-A (56) and (7″*R*,8″*R*)-8″-syringaresinol-4″-hydroxy-3″,5″-dimethoxyphenyl-7″,9″-propanediol (57). Both compounds possess a polymethoxy-substituted lignan core skeleton, which is highly consistent with the active structural features of known anti-AD lignans. In a corticosterone-induced PC12 cell injury model, which mimics the pathology of neuronal damage under high-stress conditions in the AD brain, compound 56 exhibited significant neuroprotective activity. At a concentration of 10 μM, it increased the neuronal survival rate from 45.50 ± 2.23% to 65.98 ± 1.29%, with a protective effect comparable to that of the positive control drug desipramine. Compound 57 exhibited moderate activity, increasing the neuronal survival rate to 58.19 ± 2.94% at a concentration of 10 μM. Both compounds directly protect neurons from stress-induced injury, which is highly relevant to the core pathological processes of neuronal apoptosis and hyperactive stress responses in the course of AD. Among them, compound 57, as a syringaresinol derivative, retains the activity advantages of classical anti-AD lignans, while compound 56, with its higher activity, provides a new direction for the structural optimization and modification of anti-AD lignans. These compounds expand the structural diversity and activity evidence for anti-AD lignans, offering an important experimental basis for the development of AD neuroprotective drugs targeting neuronal stress-induced injury [93]. The effects of the compounds are summarized in Table 2.

## 4. Pharmacokinetics, Bioavailability, and Druggability Challenges of Lignans Against Alzheimer’s Disease

Natural lignans have demonstrated tremendous anti-AD potential due to their advantages such as multi-target effects, anti-aging properties, and neuroprotection. However, their oral absorption, blood–brain barrier penetration, tissue distribution, metabolic characteristics, and in vivo exposure levels constitute the core bottlenecks determining their central efficacy and clinical translation. Grounded in empirical pharmacokinetic data, including UHPLC-MS/MS quantitative pharmacokinetics, pharmacokinetic differences between AD pathological states and normal states, gut microbiota-mediated prodrug activation, and brain/plasma concentration ratios, this chapter systematically reveals the in vivo fate of lignans, providing a translational basis for targeted delivery, structural optimization, and precision dosing.

### 4.1. Oral Absorption and Bioavailability: AD Pathological State Significantly Alters Systemic Exposure

Most lignans are lipophilic, poorly water-soluble polyphenols, and their oral absorption occurs primarily in the duodenum and jejunum, exhibiting common characteristics such as dissolution-limited absorption, a strong first-pass effect, and low bioavailability. For dibenzocyclooctadiene-type lignans (e.g., schisandrin, schisandrin B, and gomisins), the oral Tmax ranges from 1 to 4 h. Compared with normal rats, the AUC_0−t_ and Cmax are significantly increased and the clearance rate is slowed in AD model rats, suggesting that the AD pathological state can markedly enhance the systemic exposure of lignans [152]. All lignans demonstrate high gastrointestinal (GI) absorption, supporting their potential for oral administration. Most compounds, including schisandrin A, schisandrin B, and gomisin A, can cross the BBB, highlighting their potential for neurological applications. However, some lignans, such as gomisin B, gomisin C, and gomisin G, lack BBB permeability, which may limit their use as central nervous system targets. Additionally, some lignans are substrates or inhibitors of key cytochrome P450 enzymes, including CYP2D6 and CYP2C9, and may compete metabolically with other drugs. Such competition could lead to increased plasma and tissue drug concentrations, thereby raising the risk of toxicity [153]. Bisepoxylignans (such as sesamin, pinoresinol, and syringaresinol) exhibit extremely poor oral absorption in their prototype forms. They must rely on gut microbiota-mediated conversion into enterodiol and enterolactone to enter the bloodstream and exert their effects, thus representing typical natural prodrugs [85]. The half-life of their active metabolites can reach 4.4 ± 1.3 h, resulting in more stable exposure and more sustained action [141]. Biphenyl-type lignans (such as magnolol and honokiol) exhibit high membrane permeability but extremely low water solubility. Their rapid metabolism in the liver and intestine significantly limits the maintenance of effective concentrations in the brain [154].

In summary, low aqueous solubility, extensive first-pass metabolism, rapid systemic clearance, and enterohepatic recirculation are the four core factors contributing to the low bioavailability of lignans. Furthermore, the finding that AD pathological states can enhance the in vivo exposure of lignans offers a novel perspective for precision dosing and therapeutic efficacy improvement.

### 4.2. Tissue Distribution and Blood–Brain Barrier Penetration: Scaffold Structure Determines Brain Entry Efficiency

BBB penetration is a prerequisite for the druggability of anti-AD drugs. Due to their moderate lipophilicity, suitable molecular weight, and neutral or weak basic nature, lignans can cross the BBB to varying degrees, exhibiting a clear structure–activity relationship in which the scaffold determines the penetration efficiency [154]. Dibenzocyclooctadiene-type lignans exhibit the strongest BBB penetration capacity, with brain/plasma concentration ratios reaching 0.3–0.8, and are well distributed in cognition-critical regions such as the hippocampus and cortex, making them the most thoroughly studied category in anti-AD pharmacokinetic research to date [152]. In contrast, bisepoxylignans in their prototype form exhibit weak BBB penetration capacity; however, their gut microbiota-derived metabolite enterolactone can efficiently enter the brain and represents the key form responsible for exerting central neuroprotective effects [79,85]. Certain lignans are substrates of efflux transporters such as P-gp, BCRP, and OATP, and the efflux action can further reduce their steady-state concentrations in the brain [154]. These lines of evidence indicate that lignans possess clear brain-targeting potential; however, insufficient brain exposure remains a common limitation that must be overcome through delivery system optimization or structural modification.

### 4.3. Metabolic Characteristics: Dual-Pathway Regulation by Hepatic Metabolism and Gut Microbiota

The in vivo metabolism of lignans follows a dual-pathway paradigm: hepatic phase I and II biotransformation, coupled with colonic microbiota-mediated metabolic activation. This bifurcated metabolic fate critically governs the magnitude, duration, and interindividual variability in their pharmacological effects. Hepatic metabolism: Phase I metabolism is primarily characterized by hydroxylation, demethylation, and dehydrogenation catalyzed by CYP3A4 and CYP2C9; phase II metabolism mainly involves glucuronidation and sulfation as the major inactivation pathways, with the metabolites being rapidly cleared via bile or the kidneys [153,153]. Gut microbiota-mediated metabolism: This is critically important for bisepoxylignans and dibenzylbutane-type lignans. Through reactions such as hydrolysis, demethylation, dehydroxylation, and reduction, low-activity prototypes can be converted into highly active, high-bioavailability enterolactone and enterodiol, which serve as the core molecular basis for exerting effects via the gut–brain axis [85,152]. Species differences: Although the metabolic profiles are similar among rats, mice, and humans, significant differences exist in clearance rates; therefore, caution is warranted when extrapolating to clinical settings [155].

### 4.4. Key Pharmacokinetic Parameters

Schisandra-type lignans: *T_ma_*_x_ = 1–4 h, *t*_1/2_ = 1–6 h, with significantly higher AUC in AD rats than in normal rats [152]. Sesamin and its metabolites: poor absorption of the prototype, *t*_1/2_ < 6 h, with enterolactone serving as the centrally active form [141]. Magnolol/Honokiol: can rapidly enter the brain, with a shorter half-life in the brain than in plasma, suggesting rapid metabolism or efflux in the brain [155]. Enterolactone: *t*_1/2_ = 4.4 ± 1.3 h, with stable exposure; individual variability is primarily attributed to differences in gut microbiota composition [85].

### 4.5. Strategies for Improving Bioavailability and Brain Targeting

Based on real pharmacokinetic bottlenecks, the current optimization strategies with the greatest translational value include: structural modifications such as hydroxyl etherification, esterification, glycosylation, and prodrug design to improve solubility and metabolic stability [155]. Brain-targeted delivery systems: nanoparticles, liposomes, nanoemulsions, and solid dispersions, which significantly enhance oral absorption and BBB penetration efficiency [154]. Inhibition of efflux transporters: coadministration with P-gp inhibitors or design of non-P-gp substrate derivatives to enhance brain retention [153]. Gut microbiota modulation: targeted enrichment of bacterial strains with high efficiency in converting lignans to enhance prodrug activation [152]. Formulation optimization: self-microemulsifying drug delivery systems (SMEDDSs), enteric-coated formulations, and phospholipid complexes to improve dissolution and absorption [155]. These strategies can overcome the druggability bottlenecks of natural products, enabling lignans to be upgraded from “dietary components” to “anti-AD drug candidates,” representing an innovative direction with substantial clinical translational value.

Lignans possess unique translational advantages, including the ability to cross the BBB, elevated exposure under AD conditions, multi-target anti-AD effects, and gut microbiota-mediated prodrug activation. However, low oral bioavailability, inadequate brain exposure, rapid systemic metabolism, and marked interindividual variability remain core bottlenecks. Based on real quantitative pharmacokinetic data, comparisons between AD and normal animals, BBB penetration patterns, and microbiota-mediated prodrug mechanisms, this review establishes, for the first time, a systematic framework linking the lignan scaffold to AD-ME and central pharmacodynamics, providing scientific support for the development of a new generation of lignan-based drug candidates with high bioavailability, brain targeting, and dual anti-aging/anti-AD functionality. Pharmacokinetic and bioavailability studies are not only crucial for elucidating their in vivo mechanisms of action but also serve as a core bridge linking pharmacological activity to clinical translation. Future research should strengthen ADME–pharmacodynamics–target association studies, and integrate structural optimization, brain-targeted delivery, and gut microbiota modulation to systematically enhance the druggability of lignans, thereby advancing them from the laboratory toward clinical application in anti-AD therapy.

## 5. Current Status and Challenges of Lignans in Anti-Alzheimer’s Disease Research

### 5.1. Current Research Status

To date, significant progress has been made in research on lignans against AD, as mainly reflected in the following aspects: A diverse library of active compounds has been established. Over 90 lignan monomers with anti-AD activity have been isolated and identified from various medicinal plants and medicine–food homologous plants, including *Schisandra chinensis*, *Eucommia ulmoides*, *Sesamum indicum*, *Magnolia officinalis*, *and Arctium lappa* (as shown in Table 1 of this review). Based on their characteristic carbon skeletons, these compounds can be classified into nine major categories, including dibenzocyclooctene-type, tetrahydrofuran-type, diepoxy-type, and benzofuran-type lignans, providing rich structural diversity for drug screening. Preliminary elucidation of the multi-target pharmacological mechanism network: Studies have confirmed that lignans can systematically intervene in the complex pathological network of AD. Their mechanisms of action have evolved from early single-target effects (e.g., inhibiting AChE) to encompass multiple dimensions, including inhibiting Aβ aggregation and promoting its clearance (e.g., schisandrin A regulating autophagy), antagonizing Tau protein hyperphosphorylation (e.g., arctigenin regulating the PI3K/Akt/GSK3β pathway), anti-oxidative stress (e.g., (+)-lariciresinol activating the Nrf2 pathway), inhibiting neuroinflammation (e.g., gomisin A blocking the NF-κB/MAPK pathway), protecting mitochondrial function (e.g., honokiol activating SIRT3), inhibiting ferroptosis (e.g., schisandrin B regulating the Gsk3β/Nrf2/Gpx4 axis), and regulating gut microbiota (e.g., prodrug conversion of diepoxy-type lignans). Preliminary summary of structure–activity relationship (SAR) rules: Based on the activity differences of lignans with different skeletons, researchers have preliminarily summarized the SAR rules. For example, the “cage-like” structure of dibenzocyclooctene-type lignans is key for efficient brain entry and targeting the Nrf2 pathway; the trans double bond in the C3 side chain of benzofuran neolignans is essential for their neuroprotective activity; and the activity of diepoxy-type lignans is entirely dependent on their metabolism by gut microbiota into enterolactone. Initial progress in pharmacokinetics and druggability research: Studies have revealed the in vivo processes of lignans. For instance, the AD pathological state significantly increases the plasma concentration of Schisandra lignans; diepoxy-type lignans are typical natural prodrugs; and the BBB permeability of lignans is determined by their core skeleton. These findings have pointed the way for subsequent structural optimization and formulation development.

### 5.2. Problems

There are certain limitations at the research level. Despite the promising prospects, the following critical translational barriers remain before lignans can truly become clinically available anti-AD drugs: Insufficient clinical translation and low level of evidence: This is the most critical issue at present. The vast majority of studies remain at the level of in vitro cellular assays and rodent animal models. There is currently a severe lack of well-designed, large-scale, multicenter, longitudinal clinical trials to verify the actual efficacy, safety, and tolerability of lignans in AD patients. Existing human studies are mostly epidemiological association studies between dietary intake (e.g., flaxseed lignans) and cognitive health, which cannot prove causality. Vague “network synergy” and “core targets” of the mechanism of action: Although it has been found that lignans can regulate multiple signaling pathways such as Nrf2, NF-κB, and SIRT3, the crosstalk and synergistic regulatory mechanisms among these pathways remain unclear. For example, how lignans precisely regulate oxidative stress while inhibiting neuroinflammation, and which of these effects is the cause and which is the consequence remain a “black box”. Most studies stop at verifying that “a certain compound regulates a certain pathway,” lacking precise identification and validation of upstream direct targets (e.g., specific receptors, enzymes, or transcription factors).

Prominent druggability limitations and inadequate brain exposure constitute a critical translational choke point for lignan-based anti-AD therapeutics. Low oral bioavailability: most lignans (e.g., schisandrin B, honokiol) have extremely poor water solubility and are easily metabolized rapidly by hepatic CYP450 enzyme systems (strong first-pass effect), resulting in generally low oral bioavailability. Limited brain targeting: Although some lignans (e.g., schisandrin) can penetrate the BBB, the brain-to-plasma concentration ratio is typically low, meaning that most of the drug remains in the periphery. The action of efflux transporters such as P-glycoprotein further reduces drug accumulation in the brain. Rapid metabolism and individual differences: Most lignans have a short half-life in vivo (1–6 h), necessitating frequent dosing. In particular, the efficacy of diepoxy-type lignans is highly dependent on the ability of an individual’s gut microbiota to convert them into active metabolites (e.g., enterolactone), leading to substantial inter-individual variability and severely affecting the stability and predictability of therapeutic efficacy. Fragmented and unsystematic structure–activity relationship (SAR) research: Existing SAR studies are mostly based on scattered activity data and have failed to establish a “panoramic” correlation between “core skeleton-substituents-targets-in vivo behavior” For example, while a specific methoxy substitution may enhance in vitro antioxidant activity, how it influences water solubility, metabolic stability, or BBB penetration remains unknown. Such multi-dimensional SAR research is extremely lacking, making true “rational drug design” impossible. Insufficient safety evaluation, particularly lack of long-term data: Most in vivo studies only involve short-term observations in mouse models, and reports of “no obvious toxicity” are overly general. There is a lack of systematic, long-term toxicological and safety evaluations targeting the potential issues in the elderly population (the high-risk group for AD), such as decreased liver and kidney function and drug–drug interactions. The safety of structurally modified lignan derivatives (e.g., esterified and glycosylated products) is even more unknown. Lag in upstream preparation processes and downstream drug delivery research: Most highly active lignans have low content in plants, and their extraction and isolation processes are complex and costly, making large-scale, high-purity production difficult to achieve. Furthermore, although a few studies have attempted delivery strategies such as nanoformulations and liposomes to address their druggability shortcomings, most of these are still in the exploratory stage, lacking mature and industrializable formulation solutions.

## 6. Outlook

Moving forward, to propel lignans from the laboratory to clinical treatment of AD, a systematic paradigm shift in research is urgently needed, achieving breakthroughs across three dimensions: mechanistic innovation, technological innovation, and translational innovation.

Deepening mechanistic research: from “pathway description” to “target validation” and “network regulation”. Precisely identifying direct targets of action: Future research should go beyond descriptive pathway activation/inhibition and employ high-throughput technologies such as chemical proteomics, cellular thermal shift assay (CETSA), and drug affinity responsive target stability (DARTS) to directly identify the binding proteins of lignans within cells. For example, whether schisandrin B directly binds to GSK3β or acts on its upstream regulatory molecules should be determined. Dissecting the network mechanisms of multi-pathway synergy: system pharmacology and network biology approaches should be utilized to integrate multi-target regulatory data of lignans on Aβ, Tau, inflammation, oxidative stress, and other processes, constructing a multi-level “compound–target–pathway–phenotype” regulatory network. The dynamic interactions among core hubs such as Nrf2/NF-κB/SIRT3 should be investigated, and how lignans synergistically restore molecular network homeostasis under aging and AD conditions should be elucidated. Focusing on cutting-edge cross-mechanisms: the novel roles of lignans in the crosstalk between ferroptosis and autophagy, neurovascular unit protection, the gut–brain axis (e.g., regulating microbial metabolites such as short-chain fatty acids and tryptophan metabolism), and epigenetic regulation (e.g., miRNA, DNA methylation) should be explored in depth.

### 6.1. Overcoming Druggability Bottlenecks: From “Natural Products” to “Drug Candidates”

Rational structural modification guided by multidimensional SAR: A multi-dimensional quantitative structure–activity relationship (3D-QSAR) model encompassing “core skeleton-substituents-target activity-ADME-toxicity” should be established. Through rational design, hydroxyl and methoxy groups (e.g., via esterification, etherification, or heteroatom introduction) should be precisely modified to significantly improve metabolic stability, water solubility, or reduce efflux rates while maintaining activity. Development of brain-targeting advanced formulations: modern pharmaceutics techniques should be utilized to construct delivery systems that can efficiently encapsulate lignans and cross the BBB. Nano-enabled brain-targeted delivery: biomimetic nanoparticles (e.g., modified with ApoE or transferrin), exosomes, polymeric micelles, etc., to achieve precise brain delivery of lignans should be developed. Prodrug design: to address the issue of excessively rapid metabolism, prodrugs that can release the active parent drug under the action of brain-specific enzymes (e.g., neuron-specific enolase) should be designed. Regulating gut microbiota to optimize prodrug effects: For lignans whose activity depends on microbial metabolism (e.g., diepoxy-type lignans), individual differences can be eliminated by co-administering prebiotics/probiotics, designing microbiota-responsive oral formulations (e.g., colon-targeted capsules), or directly identifying and producing their final active metabolites (e.g., enterolactone) as alternative drugs.

### 6.2. Accelerating Clinical Translation: From “Incremental Research” to “Paradigm Innovation”

Establishing a distinct clinical positioning: Given that lignans possess the dual characteristics of delaying aging and multi-target regulation, their clinical positioning should not be to replace existing symptomatic drugs (e.g., donepezil), but rather to focus on: early intervention by conducting long-term, prospective preventive clinical trials in populations with mild cognitive impairment (MCI) or asymptomatic individuals at high risk (e.g., APOEε4 carriers) to verify their efficacy in delaying disease onset; combination therapy by exploring synergistic treatment regimens combining lignans with low doses of approved drugs (e.g., donepezil, memantine) to achieve “ toxicity reduction and efficacy enhancement”; and advancing high-quality clinical research by designing and conducting multicenter, randomized, double-blind, placebo-controlled Phase II/III clinical trials that strictly adhere to GCP guidelines. In addition to traditional cognitive scales (e.g., ADAS-Cog), cerebrospinal fluid/blood biomarkers (Aβ_42/40_, p-Tau, NfL), neuroimaging indicators (PET assessment of Aβ/Tau deposition), and gut metagenomics should be incorporated as secondary endpoints to provide multi-dimensional evidence of efficacy. Biomarker-based patient stratification strategies should be developed: By detecting individual gut microbiota composition (for diepoxy-type lignans) or drug-metabolizing enzyme genotypes (CYP450 polymorphisms), responder populations should be screened to achieve personalized treatment, thereby improving clinical trial success rates and long-term outcomes. A comprehensive age-relevant safety evaluation framework should be established: Before advancing to clinical trials, long-term toxicology studies (at least 6–9 months) compliant with GLP regulations must be completed, with a focus on the effects on liver and kidney function, as well as cognitive function (unexpected effects), in aged animals. Additionally, metabolic interactions with commonly used clinical AD drugs and other cardiovascular and antidiabetic medications should be systematically assessed.

### 6.3. Conclusions: Constructing a “Drug–Microbiota–Host” Trinity Intervention System

In summary, as a class of highly promising natural anti-aging and anti-AD lead compounds, the future development of lignans should be pursued as a systematic engineering endeavor. We should move beyond the traditional R&D model of single compound and single target, and instead construct a four-in-one R&D and application system integrating “precision chemistry (structural optimization)-advanced formulations (brain-targeted delivery)-host modulation (patient stratification)-microbiota intervention (individualized prodrug activation)”. Through this multi-dimensional, interdisciplinary strategy, it is expected that the existing translational bottlenecks will ultimately be overcome, transforming lignans, a gift from nature, into a new generation of multi-target drugs or functional interventions capable of delaying or even halting the progression of aging-driven AD, thereby providing Chinese wisdom and innovative solutions to the increasingly severe global challenge of cognitive health.

## Figures and Tables

**Table 2:** Pharmacological effects and active doses of lignans in the treatment of Alzheimer’s disease.

Activity	Compounds	Active Dose	Model	Source	Reference
Anti-inflammatory activity	19, 20, 21, 22, 23, 24, 25, 26, 27, 28	IC_50_: 1.91–72.05 µM	In vitro, inhibitory effects on LPS-induced NO release in BV2 microglia.	The fruits of *S. chinensis*	[78]
	29	Test dose: 3–100 μM	In vitro, inhibition of LPS-induced markers in microglia cells.	The fruits of *S. chinensis* (Turcz.) Baill.	[144]
	31	Test dose: 5, 10, 15 µg/mL	In vitro, Aβ_25−35_-induced human neuroblastoma SH-SY5Y and SK-N-SH cell inflammatory cytokine (IL-6, IL-1β, TNF-α) levels by ELISA.	The fruits of *S. chinensis* (Turcz.) Baill.	[143,145]
	32	Test dose: 5, 10, 20 µM Test dose: 5, 10, 20 µM Test dose: 5, 10, 20 µM Test dose: 5, 10, 20 µM	In vitro, LPS-induced rat primary microglia-enriched cultures inflammatory mediator production by NO assay, ELISA assay and RT-PCR. In vitro, LPS-induced rat primary neuron–microglia co-cultures neuronal cell death by MTT assay and TUNEL assay. In vitro, LPS-induced rat primary cortical neuron-enriched cultures viability by MTT assay. In vitro, LPS-induced rat primary microglia NF-κB nuclear translocation by immunofluorescence assay and Western blotting assay.	The fruits of *Schisandra chinensis* Turcz. (Baill.)	[148]
	39	Effective dose: 30 mg/kg/day (P.O., 8 consecutive weeks)	In vivo, effects on STZ-induced diabetic rats, anti-inflammatory (inhibits NF-κB, TNF-α, IL-1β, IL-6; increases IL-10 in cerebral cortex and hippocampus). In vitro, effects on LPS-stimulated cells, (suppresses p38 mitogen-activated protein kinase, NF-κB; reduces cytokine production).	Sesame seed	[86]
	40	Test dose: 25, 50 mg/kg, P.O.	In vivo, (-)-sesamin alleviates chronic electric foot shock-induced spatial and habit learning memory deficits in ICR mice by modulating NMDA receptor phosphorylation, ERK1/2-CREB signaling, and striatal dopamine levels.	*Asiasari Radix*	[87]
	47	Test doses: 10–50 µg/mL	In vitro, inhibitory effects on LPS-induced BV2 microglia cells, test for changes in TNF-α, IL-6, IL-1β and iNOS (mRNAs and proteins).	The fruits of the *Forsythia suspensa* (Thunb.)	[89]
	14	Test doses: 12.5, 25, 50 μM IC_50_: 20–30 μM	In vitro, inhibitory effects on LPS-induced BV2 microglia cells, test for changes for TNF-α and IL-6.	The fruits of *Arctium lappa* L.	[150]
	52, 53	Test dose: 5 μM, 10 μM, 20 μM.	In vitro, inhibitory effects on LPS-induced BV2 microglia cells, test for changes in NO, PGE_2_, TNF-α, IL-6, MCP-1, iNOS and COX-2, as well as p-ERK, p-JNK, p-P38, p-IκBα, p-p65 and p65 nuclear translocation.	*Artemisia mongolica*	[91]
	15	Effective doses: 5, 10, 20 mg/kg	In vivo, effects on LPS-induced neuroinflammatory male C57BL/6 mice, anti-inflammatory (inhibits MAPK/NF-κB pathway, NLRP3 inflammasome, NO, PGE_2_, TNF-α, IL-1β, IL-6, iNOS, COX-2 in hippocampus and serum).	*Eleutherococcus henryi Oliv*	[77]
	14	Test doses: 12.5, 25, 50 μM IC_50_: 20–30 μM	In vitro, inhibitory effects on LPS-induced BV2 microglia cells, test for changes for TNF-α and IL-6.	The fruits of *Arctium lappa* L.	[150]
Antioxidant effect	29	Test dose: 10–100 µg/mL	In vitro, in LPS-stimulated N9 microglial cells, exerted inhibitory effect on the increased expression of gp91phox.	The fruits of *S. chinensis* (Turcz.) Baill.	[144]
	30	Test dose: 12.5 μM, 25 μM, 50 μM.	In vitro, protective effects on H_2_O_2_-induced SH-SY5Y/APP695swe cells, test for changes in Nrf2, p−GSK3β Ser9/GSK3β, NQO1 and HO-1.	The fruits of *Schisandra chinensis* Turcz. (Baill.)	[142]
	31	Test dose: 5, 10, 15 µg/mL	In vitro, Aβ25−35-induced human neuroblastoma SH-SY5Y and SK-N-SH cell oxidative stress (SOD, GSH, MDA) by corresponding kits.	The fruits of *S. chinensis* (Turcz.) Baill.	[79,143]
	42, 43, 44	Test dose: 6.25–25 μM	In vitro, H_2_O_2_-induced PC-12 cells.	*E. ulmoides leaves*	[84]
	93	Test dose: 3–30 μM Test dose: 3–30 μM Test dose: 30 μM	In vitro, protective effects on HepG2 hepatocyte cells, test for changes in GCL, NQO1 mRNA and protein levels. In vitro, activation of Nrf2-ARE pathway in HepG2 cells, test for luciferase activity. In vitro, regulation of PKCd-GSK3β pathway in HepG2 cells, test for phosphorylation of PKCd, GSK3β and Nrf2.	*Saururus chinensis*	[138]
	34	Test dose: 5,10 μM	In vitro, protective effects on Aβ oligomer-induced primary hippocampal neurons, test for changes in cell viability, mitochondrial function (ATP production, ROS levels, mitochondrial membrane potential) and neuronal apoptosis.	The barks of *Magnolia officinalis*	[151]
	72	EC_50_: 1.86 μg/mL, 0.59 μg/mL, 16.80 μg/mL	In vitro, calculation of DPPH, O^2-^ and OH free radical scavenging capacity.	*Fructus Cannabis* of *Bama*	[109]
	49, 50, 51, 77, 78	Test dose: 25 μM	In vitro, effects on enzyme activities in H_2_O_2_-treated PC12 cells.	*E. ulmoides* leaves	[84]
	54, 55	Test dose: 5 μM, 10 μM, 30 μM, 50 μM	In vitro, L-glutamate-induced mouse hippocampal HT22 cell oxidative stress and neurotoxicity (cell viability) by WST assay.	*Osmanthus fragrans* var. *aurantiacus*	[92]
	67	Test dose: 6.125 μM, 12.5 μM, 25 μM, 50 μM	In vitro, DPPH/ABTS/superoxide/hydroxyl radical scavenging assays for antioxidant capacity evaluation.	*Rubia philippinensis*	[97]
	35, 36	Test dose: 5 μM, 10 μM, 30 μM, 50 μM	In vitro, enhances cell viability of L-glutamate-induced HT22 hippocampal cells/HT22 cells, test for cell viability via WST assay.	*Magnolia officinalis*	[83]
	1, 2, 3, 11, 12, 13, 79	Test dose: 2 μM, 5 μM, 10 μM	In vitro, enhances cell viability of Aβ_25−35_-induced SH-SY5Y cells/SH-SY5Y cells, test for cell viability via MTT assay.	*Schisandra bicolo* *var. tuberculata*	[72]
	85	IC_50_: 3.08 μM	In vitro, enhances cell viability of 6-OHDA-induced SH-SY5Y cells/SH-SY5Y cells, test for cell viability via MTT assay.	*Magnolia biondii Pamp*	[133]
	86	IC_50_: 6.12 μM	In vitro, enhances cell viability of 6-OHDA-induced SH-SY5Y cells/ SH-SY5Y cells, test for cell viability via MTT assay.	*Magnolia biondii Pamp*	[133]
	83, 84	Test doses: 5, 10, 20, 40 μM	In vitro, effects on glutamate-induced oxidative injury in SH-SY5Y cells, neuroprotective (activates Nrf2/HO-1 pathway, promotes BDNF expression, increases SOD activity, reduces ROS and LDH levels).	*Curculigo capitulata*	[132]
Anticholinergic effect	32	Test dose: 10, 25, 50 mg/kg.	In vivo, preventive effects on scopolamine-induced memory deficits in mice.	The fruits of *Schisandra chinensis* Turcz. (Baill.)	[148]
	8	IC_50_: 0.67 μM	In vitro, test inhibitory capacity of AChE with ATCI as substrate.	The leaves of *Isatis indigotica* *Fortune*	[94]
	58	IC_50_: 992 μM	Ex vivo, evaluate modulatory effect of cubebin on brain AChE levels in scopolamine-induced amnesic mice.	The fruits of *Piper cubeba*	[94]
	48	(IC_50_: 85.03 ± 4.26 nM)1.64-fold increase	In vitro, inhibitory effects on AChE enzyme and telomerase activation in normal melanocyte (HFB4) cell line, test for AChE inhibition and telomerase activation.	Aerial parts of *Anisacanthus virgularis* (Salisb.) *Nees*	[90]
Gut microbiota-modulating effect	46	/	Intestinal flora.	*Rye*, whole grain flour	[85]
	37	/	Intestinal flora.	*Olive oil*	[85]
	38	/	Intestinal flora.	Sesame seed	[85]
	41	/	Intestinal flora.	Sesame seed	[85]
Neuroprotective effect	29	Test dose: 3–100 μM	In vitro, protective effects on LPS-activated microglia-conditioned media-induced SH-SY5Y neuroblastoma cells, primary rat cortical neurons and primary rat hippocampal neurons, test for changes for cell viability.	The fruits of *S. chinensis* (Turcz.) Baill.	[144]
	10	Effective dose: 30 μM, 0.01 μM	In vitro, neurite-outgrowth promotion assay in NGF-differentiated PC12 cells, and neurite-outgrowth promotion and neuroprotection assays in primary cultured rat cortical neurons.	The roots of *Aristolochia contorta*	[75]
	58, 59, 60, 61, 62	Test dose: 12.5, 25, 50 μM	In vitro, H_2_O_2_-induced human neuroblastoma SH-SY5Y cell injury model.	The leaves of *Isatis indigotica Fortune*	[94]
	88	Test dose: 10 μM	In vitro, neuroprotective effects against glutamate-induced PC12 cell injury, test for changes in cell viability.	The pseudobulbs of *Bletilla striata*	[136]
	92	Test dose: 10 μM, 2 μM	In vitro, neuroprotective effects against glutamate -induced PC12 cell injury, test for changes in cell viability.	The pseudobulbs of *Bletilla striata*	[136]
	76	Test dose: 10–20 μM	In vitro, inhibitory effects on LPS- induced BV2 microglia cells, test for changes for TNF-α and IL-6.	*Hemp* seed	[147]
	87	Test dose: 0, 5, 10, 20, 40 and 80 μM	In vitro, NMDA-induced human neuroblastoma SH-SY5Y cell viability by CCK8 assay.	*Lycium barbarum*	[134]
	68, 69, 70, 71, 72, 73, 74, 75	IC_50_: 8.28 ± 2.29 μM, 40.39 ± 2.97 μM, 27.62 ± 2.52 μM, 36.9 ± 2.34 μM, 18.96 ± 4.47 μM, 4.26 ± 0.93 μM, 40.68 ± 5.36 μM, 12.13 ± 1.00 μM	In vitro, inhibitory effects on LPS- induced NO production in BV2 microglia cells.	Stems of *Piperhancei*	[102]
	80, 81, 82	Test dose: 1 μM, 10 μM, 30 μM	In vitro, NMDA-induced primary cortical neuron damage.	*Adelostemma gracillimum*	[131]
	56, 57	Test dose: 10μM	In vitro, enhances cell viability of corticosterone-induced PC12 cells/PC12 cells, test for cell viability via MTT assay.	*Aconiti lateralis Radix Praeparata*	[93]
	5, 6, 16, 17, 18	Test dose: 10 μM	In vitro, effects on SNP-induced neurotoxicity in PC12 cells.	*Wikstroemia alternifolia*	[73]
	89, 90, 91	Effective doses: 2.5, 5, 10 μM	In vitro, effects on SNP-induced neurotoxicity in PC12 cells, neuroprotective (inhibits oxidative stress-mediated neuronal apoptosis).	*Phyllanthodendron breynioides*	[137]
Anti-Aβ aggregation effect	63, 64	Test dose: 20 μM	In vitro, inhibitory effects on self-induced Aβ_1−42_ aggregation, test for changes for inhibitory rate and binding interactions with Aβ_1−42_ residues	Dried and powdered seeds of *Prunus tomentosa* Thunb.	[95]
	65, 66	Test dose: 20 μM	In vitro, inhibitory effects on Aβ_42_ aggregation, test for changes in anti-Aβ aggregation activity.	The twigs and leaves of *Pithecellobium clypearia Benth*	[96]
	4	Test dose: 5 μM,10 μM	In vitro, enhances cell viability of Aβ_25−35_-induced SH-SY5Y cells/SH-SY5Y cells, test for cell viability via MTT assay.	*Schisandra bicolo* var. *tuberculata*	[72]
Anti-bacterial infection effect	8, 9	MIC: 50 μg/mL	Porphyromonas gingivalis suspension.	The synthesized compounds	[74]
Neuromodulatory effect	45	Test dose: 10–500 μM (IC_50_: 90 μM) Test dose: 50 μM	In vitro, inhibitory effects on excitatory synaptic transmission in mouse hippocampal SC-CA1 synapses. In vitro, suppression of picrotoxin-induced epileptiform activity in mouse hippocampal slices.	*Panax ginseng berries*	[88]
Autophagic effect	33	Test dose: 2μM, 5μM Test dose: 5 μM, 10 μM	In vitro, promotes autophagy and inhibits apoptosis in AβO-induced BV2 cells, test for changes in autophagy-related proteins (Beclin-1, LC3II, p62) and apoptosis-related proteins (cleaved-caspase-9, Bax, Bcl-2. In vitro, promotes autophagy and inhibits apoptosis in AβO-induced N2a cells, test for changes in autophagy-related proteins (Beclin-1, LC3II, p62) and apoptosis-related proteins (cleaved-caspase-9, Bax, Bcl-2).	*Magnolia officinalis*	[81]
Cytoprotective effect	33	Test dose: 2 μM, 5μM, 10 μM	In vitro, enhances cell viability of AβO-induced BV2 cells, test for cell viability via MTT assay.	*Magnolia officinalis*	[81]

## Data Availability

Data sharing is not applicable to this article as no new data were created or analyzed.

## Abbreviations

The following abbreviations are used in this manuscript:

AD	Alzheimer’s disease
Aβ	Amyloid-β
NFTs	Neurofibrillary tangles
APP	Amyloid precursor protein
AICD	APP intracellular domain
APOE	Apolipoprotein E
AChE	Acetylcholinesterase
MAP	Microtubule-associated protein
MTs	Microtubules
NF-κB	Nuclear factor kappa-B
ACh	Acetylcholine
OS	Oxidative stress
ROS	Reactive oxygen species
ER	Endoplasmic reticulum
HO-1	Heme oxygenase-1
NQO-1	NAD(P)H:quinone oxidoreductase 1
CAT	Catalase
SOD	Superoxide dismutase
GPx	Glutathione peroxidase
SNP	Sodium nitroprusside
NO	Nitric oxide
MAPK	Mitogen-activated protein kinase
Nrf2	Nuclear factor erythroid 2-related factor 2
Keap1	Kelch-like ECH-associated protein 1
NAC	N-acetylcysteine
BDNF	Brain-derived neurotrophic factor
ATP	Adenosine triphosphate
GSH-Px	Glutathione peroxidase
GSH	Glutathione
MDA	Malondialdehyde
GSSG	Oxidized glutathione
NADPH	Nicotinamide adenine dinucleotide phosphate
NMDA	N-methyl-D-aspartate
LPS	Lipopolysaccharide
PGE_2_	Prostaglandin E_2_
MCP-1	Monocyte chemoattractant protein-1
p-IκBα	Phosphorylated inhibitor of κB α
p-p65	Phosphorylated p65
PAT	Passive avoidance task
MWM	Morris water maze
IC_50_	Half-maximal inhibitory concentration
EF	Electric foot shock
ERK1/2	Extracellular signal-regulated kinase
CREB	CAMP response element-binding protein
Gsk3β	Glycogen synthase kinase 3β
FSP1	Ferroptosis suppressor protein 1
INDO	Inhibiting indomethacin
GI	Gastrointestinal
BBB	Blood–brain barrier
SMEDDS	Self-microemulsifying drug delivery systems
SAR	Structure–activity relationship
CETSA	Cellular thermal shift assay
DARTS	Drug affinity responsive target stability

## References

[1] World Health Organization (2021). Dementia [Internet].

[2] Shanmugam J.V., Duraisamy B., Simon B.C., Bhaskaran P. (2022). Alzheimer’s disease classification using pre-trained deep networks. Biomed. Signal Process. Control.

[3] Li X., Feng X., Sun X., Hou N., Han F., Liu Y. (2022). Global, regional, and national burden of Alzheimer’s disease and other dementias, 1990–2019. Front. Aging Neurosci..

[4] Abdelnour C., Agosta F., Bozzali M., Fougère B., Iwata A., Nilforooshan R., Takada L.T., Viñuela F., Traber M. (2022). Perspectives and challenges in patient stratification in Alzheimer’s disease. Alzheimers Res. Ther..

[5] Cummings J. (2021). New approaches to symptomatic treatments for Alzheimer’s disease. Mol. Neurodegener..

[6] McKhann G.M., Knopman D.S., Chertkow H., Hyman B.T., Jack C.R., Kawas C.H., Klunk W.E., Koroshetz W.J., Manly J.J., Mayeux R. (2011). The diagnosis of dementia due to Alzheimer’s disease: Recommendations from the National Institute on Aging-Alzheimer’s Association workgroups on diagnostic guidelines for Alzheimer’s disease. Alzheimers Dement..

[7] Tarawneh R., Holtzman D.M. (2012). The clinical problem of symptomatic Alzheimer disease and mild cognitive impairment. Cold Spring Harb. Perspect. Med..

[8] Knopman D.S., Amieva H., Petersen R.C., Chételat G., Holtzman D.M., Hyman B.T., Nixon R.A., Jones D.T. (2021). Alzheimer disease. Nat. Rev. Dis. Prim..

[9] Maciejewska K., Czarnecka K., Szymański P. (2021). A review of the mechanisms underlying selected comorbidities in Alzheimer’s disease. Pharmacol. Rep..

[10] Dubois B., von Arnim C.A.F., Burnie N., Bozeat S., Cummings J. (2023). Biomarkers in Alzheimer’s disease: Role in early and differential diagnosis and recognition of atypical variants. Alzheimers Res. Ther..

[11] Katabathula S. (2023). Comorbidity-driven multi-modal subtype analysis in mild cognitive impairment of Alzheimer’s disease. Alzheimers Dement..

[12] Wang S., Wang L., Qin X., Turdi S., Sun D., Culver B., Reiter R.J., Wang X., Zhou H., Ren J. (2020). ALDH2 contributes to melatonin-induced protection against APP/PS1 mutation-prompted cardiac anomalies through cGAS-STING-TBK1-mediated regulation of mitophagy. Signal Transduct. Target. Ther..

[13] Zhan X., Stamova B., Jin L.-W., DeCarli C., Phinney B., Sharp F. (2016). Lipopolysaccharide and E. Coli proteins and DNA in Alzheimer’s disease brains compared to controls. Alzheimers Dement..

[14] Heneka M.T., van der Flier W.M., Jessen F., Hoozemanns J., Thal D.R., Boche D., Brosseron F., Teunissen C., Zetterberg H., Jacobs A.H. (2025). Neuroinflammation in Alzheimer disease. Nat. Rev. Immunol..

[15] Zhou Y. (2021). Pharmacodynamic effects and molecular mechanisms of lignans from Schisandra chinensis Turcz. (Baill.). Eur. J. Pharmacol..

[16] Grodzicki W., Dziendzikowska K. (2020). The Role of Selected Bioactive Compounds in the Prevention of Alzheimer’s Disease. Antioxidants.

[17] Yin R.-H., Yu J.T., Tan L. (2015). The role of SORL1 in Alzheimer disease. Mol. Neurobiol..

[18] Association A. (2012). 2012 Alzheimer’s disease facts and figures. Alzheimers Dement..

[19] Ashrafian H., Hadi Zadeh E., Khan R.H. (2021). Review on Alzheimer’s disease: Inhibition of amyloid beta and tau tangle formation. Int. J. Biol. Macromol..

[20] Zhang H., Wei W., Zhao M., Ma L., Jiang X., Pei H., Cao Y., Li H. (2021). Interaction between Aβ and tau in the pathogenesis of Alzheimer’s disease. Int. J. Biol. Sci..

[21] Vassar R., Bennett B.D., Babu-Khan S., Kahn S., Mendiaz E.A., Denis P., Teplow D.B., Ross S., Amarante P., Loeloff R. (1999). Beta-secretase cleavage of Alzheimer’s amyloid precursor protein by the transmembrane aspartic protease BACE. Science.

[22] Hardy J.A., Higgins H. (1992). Alzheimer’s disease: The amyloid cascade hypothesis. Science.

[23] De Ferrari G.V., Canales M.A., Shin I., Weiner L.M., Silman I., Inestrosa N.C. (2001). A structural motif of acetylcholinesterase that promotes amyloid beta-peptide fibril formation. Biochemistry.

[24] Medeiros R., Baglietto-Vargas D., LaFerla F.M. (2011). The role of tau in Alzheimer’s disease and related disorders. CNS Neurosci. Ther..

[25] Violet M. (2014). Tau, Oxidative Stress, hyperthermia, DNA Damage, RNA damage, γ-H2AX, DNA Repair. Front. Cell. Neurosci..

[26] Michalicova A., Majerova P., Kovac A. (2020). Tau Protein and Its Role in Blood-Brain Barrier Dysfunction. Front. Mol. Neurosci..

[27] Bi M., Gladbach A., van Eersel J., Ittner A., Przybyla M., van Hummel A., Chua S.W., van der Hoven J., Lee W.S., Müller J. (2017). Tau exacerbates excitotoxic brain damage in an animal model of stroke. Nat. Commun..

[28] Sebastián-Serrano Á., De Diego-García L., Díaz-Hernández M. (2018). The Neurotoxic Role of Extracellular Tau Protein. Int. J. Mol. Sci..

[29] Heneka M.T., O’Banion M.K., Terwel D., Kummer M.P. (2010). Neuroinflammatory processes in Alzheimer’s disease. J. Neural Transm..

[30] Dhapola R., Hota S.S., Sarma P., Bhattacharyya A., Medhi B., Reddy D.H. (2021). Recent advances in molecular pathways and therapeutic implications targeting neuroinflammation for Alzheimer’s disease. Inflammopharmacology.

[31] Heneka M.T., van der Flier W.M., Jessen F., Hoozemanns J., Thal D.R., Boche D., Brosseron F., Teunissen C., Zetterberg H., Jacobs A.H. (2015). Neuroinflammation in Alzheimer’s disease. Lancet Neurol..

[32] Si Z.-Z., Zou C.J., Mei X., Li X.F., Luo H., Shen Y., Hu J., Li X.X., Wu L., Liu Y. (2023). Targeting neuroinflammation in Alzheimer’s disease: From mechanisms to clinical applications. Neural Regen. Res..

[33] Bekdash R. (2021). The Cholinergic System, the Adrenergic System and the Neuropathology of Alzheimer’s Disease. Int. J. Mol. Sci..

[34] Terry A.V., Buccafusco J.J. (2003). The Cholinergic Hypothesis of Age and Alzheimer’s Disease-Related Cognitive Deficits: Recent Challenges and Their Implications for Novel Drug Development. J. Pharmacol. Exp. Ther..

[35] Bartus R.T., Dean D., Beer B., Lippa L. (1982). The cholinergic hypothesis of geriatric memory dysfunction. Science.

[36] Schwarthoff S., Tischer N., Sager H., Schätz B., Rohrbach M.M., Raztsou I., Robaa D., Gaube F., Arndt H.D., Winckler T. (2021). Evaluation of γ-carboline-phenothiazine conjugates as simultaneous NMDA receptor blockers and cholinesterase inhibitors. Bioorg. Med. Chem..

[37] De Medeiros L.M., De Bastiani M.A., Rico E.P., Schonhofen P., Pfaffenseller B., Wollenhaupt-Aguiar B., Grun L., Barbé-Tuana F., Zimmer E.R., Castro M.A.A. (2019). Cholinergic differentiation of human neuroblastoma SH-SY5Y cell line and its potential use as an in vitro model for Alzheimer’s disease studies. Mol. Neurobiol..

[38] Lian W., Fang J., Xu L., Zhou W., Kang D., Xiong W., Jia H., Liu A.L., Du G.H. (2017). DL0410 Ameliorates memory and cognitive impairments induced by scopolamine via increasing cholinergic neurotransmission in mice. Molecules.

[39] Hung S.Y., Fu W.M. (2017). Drug candidates in clinical trials for Alzheimer’s disease. J. Biomed. Sci..

[40] Chen Z.-R., Huang J.-B., Yang S.-L., Hong F.-F. (2022). Role of cholinergic signaling in Alzheimer’s disease. Molecules.

[41] Jones D.P. (2006). Redefining oxidative stress. Antioxid. Redox Signal..

[42] Perluigi M., Di Domenico F., Butterfield D.A. (2024). Oxidative damage in neurodegeneration: Roles in the pathogenesis and progression of Alzheimer disease. Physiol. Rev..

[43] Markesbery W.R. (1997). Oxidative stress hypothesis in Alzheimer’s disease. Free Radic. Biol. Med..

[44] Yu N., Pasha M., Chua J.J.E. (2024). Redox changes and cellular senescence in Alzheimer’s disease. Redox Biol..

[45] Zhao Y., Zhao B. (2013). Oxidative stress and the pathogenesis of Alzheimer’s disease. Oxid. Med. Cell. Longev..

[46] Tan S., Sagara Y., Liu Y., Maher P., Schubert D. (1998). The regulation of reactive oxygen species production during programmed cell death. J. Cell Biol..

[47] Grivennikova V.G., Vinogradov A.D. (2006). Generation of superoxide by the mitochondrial complex I. Biochim. Biophys. Acta.

[48] Hirai K., Aliev G., Nunomura A., Fujioka H., Russell R., Atwood C., Johnson A., Kress Y., Vinters H., Tabaton M. (2001). Mitochondrial abnormalities in Alzheimer’s disease. J. Neurosci..

[49] Mutisya E.M., Bowling A.C., Beal M.F. (1994). Cortical cytochrome oxidase activity is reduced in Alzheimer’s disease. J. Neurochem..

[50] Reiss A.B., Gulkarov S., Jacob B., Srivastava A., Pinkhasov A., Gomolin I.H., Stecker M.M., Wisniewski T., De Leon J. (2024). Mitochondria in Alzheimer’s disease pathogenesis. Life.

[51] Jayatunga D.P.W., Hone E., Bharadwaj P., Garg M., Verdile G., Guillemin G.J., Martins R.N. (2020). Targeting mitophagy in Alzheimer’s disease. J. Alzheimers Dis..

[52] Tettevi E.J., Simpong D.L., Maina M., Adjei S., Asuming-Brempong E., Osei-Atweneboana M.Y. (2025). Antibiotic-induced gut dysbiosis modulates Alzheimer’s disease-associated gene expression and protein aggregation in 3xTg-AD mice via the gut-brain axis. Brain Behav..

[53] Bello-Corral L., Alves-Gomes L., Fernández-Fernández J.A., Fernández-García D., Casado-Verdejo I., Sánchez-Valdeón L. (2023). Implications of gut and oral microbiota in neuroinflammatory responses in Alzheimer’s disease. Life Sci..

[54] Zhang T., Gao G., Kwok L.Y., Sun Z. (2023). Gut microbiome-targeted therapies for Alzheimer’s disease. Gut Microbes.

[55] Lampe J.W., Martini M.C., Kurzer M.S., Adlercreutz H., Slavin J.L. (1994). Urinary lignan and isoflavonoid excretion in premenopausal women consuming flaxseed powder. Am. J. Clin. Nutr..

[56] Yamauchi S., Taniguchi E. (1991). Synthesis and insecticidal activity of lignan analogs (II). Biosci. Biotechnol. Biochem..

[57] Nitao J.K., Johnson K.S., Scriber J.M., Nair M.G. (1992). Magnolia virginiana neolignan compounds as chemical barriers to swallowtail butterfly host use. J. Chem. Ecol..

[58] Wang C., Mäkelä T., Hase T., Adlercreutz H., Kurzer M.S. (1994). Lignans and flavonoids inhibit aromatase enzyme in human preadipocytes. J. Steroid Biochem. Mol. Biol..

[59] Mäkelä S.I., Pylkkänen L.H., Santti R.S.S., Adlercreutz H. (1995). Dietary soybean may be antiestrogenic in male mice. J. Nutr..

[60] Pan J.-Y., Chen S.L., Yang M.H., Wu J., Sinkkonen J., Zou K. (2009). An update on lignans: Natural products and synthesis. Nat. Prod. Rep..

[61] Kawazoe K., Yutani A., Tamemoto K., Yuasa S., Shibata H., Higuti T., Takaishi Y. (2001). Phenylnaphthalene compounds from the subterranean part of Vitex rotundifolia and their antibacterial activity against methicillin-resistant Staphylococcus aureus. J. Nat. Prod..

[62] Janmanchi D., Tseng Y.P., Wang K.C., Huang R.L., Lin C.H., Yeh S.F. (2010). Synthesis and the biological evaluation of arylnaphthalene lignans as anti-hepatitis B virus agents. Bioorg. Med. Chem..

[63] McDoniel P.B., Cole J.R. (1972). Antitumor activity of Bursera schlechtendalii (Burseraceae). Isolation and structure determination of two new lignans. J. Pharm. Sci..

[64] Pelter A., Ward R.S., Satyanarayana P., Collins P. (1983). Synthesis of lignan lactones by conjugate addition of thioacetal carbanions to butenolide. J. Chem. Soc.-Perkin Trans..

[65] Wu G.R., Xu B., Yang Y.Q., Zhang X.Y., Fang K., Ma T., Wang H., Xue N.N., Chen M., Guo W.B. (2018). Synthesis and biological evaluation of podophyllotoxin derivatives as selective antitumor agents. Eur. J. Med. Chem..

[66] Weng J.-R., Ko H.H., Yeh T.L., Lin H.C., Lin C.N. (2004). Two New Arylnaphthalide Lignans and Antiplatelet Constituents from Justicia procumbens. Arch. Pharm. Pharm. Med. Chem..

[67] Kovalainen J.T., Christiaans J.A.M., Kotisaari S., Laitinen J.T., Männistö P.T., Tuomisto L., Gynther J. (1999). Synthesis and in vitro pharmacology of a series of new chiral histamine H3-receptor ligands: 2-(R and S)-Amino-3-(1H-imidazol-4(5)-yl)propyl ether derivatives. J. Med. Chem..

[68] Iwasaki T., Kondo K., Kuroda T., Moritani Y., Yamagata S., Sugiura M., Kikkawa H., Kaminuma O., Ikezawa K. (1996). Novel selective PDE IV inhibitors as antiasthmatic agents. Synthesis and biological activities of a series of 1-aryl-2,3-bis(hydroxymethyl)naphthalene lignans. J. Med. Chem..

[69] Wu S.-J., Wu T.-S. (2007). Cytotoxic Arylnaphthalene Lignans from Phyllanthus oligospermus. ChemInform.

[70] Lu H., Liu G.T. (1992). Anti-oxidant activity of dibenzocyclooctene lignans isolated from Schisandraceae. Planta Med..

[71] Hirano T., Wakasugi A., Oohara M., Oka K., Sashida Y. (1991). Suppression of mitogen-induced proliferation of human peripheral blood lymphocytes by plant lignans. Planta Med..

[72] Liu Y., Wakasugi A., Sashida Y. (2017). Neuroprotective Lignans from the Fruits of Schisandra bicolor var. tuberculata. J. Nat. Prod..

[73] Lu Q.R., Wang Y., Tang Z.Y., Chen Y., Weng H.Z., Yin S., Tang G.H. (2024). Chemical constituents of Wikstroemia alternifolia and their neuroprotective activities. Nat. Prod. Res..

[74] Rezende K.C.S., Lucarini R., Símaro G.V., Pauletti P.M., Januário A.H., Esperandim V.R., Martins C.H.G., Silva M.A., Cunha W.R., Bastos J.K. (2016). Antibacterial activity of (-)-Cubebin isolated from piper cubeba and its semisynthetic derivatives against microorganisms that cause endodontic infections. Rev. Bras. Farmacogn..

[75] Harada K., Kubo M., Horiuchi H., Ishii A., Esumi T., Hioki H., Fukuyama Y. (2015). Systematic Asymmetric Synthesis of All Diastereomers of (-)-Talaumidin and Their Neurotrophic Activity. J. Org. Chem..

[76] Li Y., Lan X., Wang S., Cui Y., Song S., Zhou H., Li Q., Dai L., Zhang J. (2022). Serial five-membered lactone ring ions in the treatment of Alzheimer’s diseases-comprehensive profiling of arctigenin metabolites and network analysis. Front. Pharmacol..

[77] Tang S., Li C., Suo Z., Xu Y., Wei K., Zhao L., Huang H., Liu X., Liu D., Li X. (2023). Neuroprotective Effects of Savinin on LPS-Induced Neuroinflammation In Vivo via Regulating MAPK/NF-κB Pathway and NLRP3 Inflammasome Activation. Molecules.

[78] Hu D., Han N., Yao X., Liu Z., Wang Y., Yang J., Yin J. (2014). Structure-activity relationship study of dibenzocyclooctadiene lignans isolated from Schisandra chinensis on lipopolysaccharide-induced microglia activation. Planta Med..

[79] Li X., Zhao X., Xu X., Mao X., Liu Z., Li H., Guo L., Bi K., Jia Y. (2014). Schisantherin A recovers Aβ-induced neurodegeneration with cognitive decline in mice. Physiol. Behav..

[80] Zeng K.W., Zhang T., Fu H., Liu G.X., Wang X.M. (2012). Schisandrin B exerts anti-neuroinflammatory activity by inhibiting the Toll-like receptor 4-dependent MyD88/IKK/NF-κB signaling pathway in lipopolysaccharide-induced microglia. Eur. J. Pharmacol..

[81] Wang X., Jia J. (2023). Magnolol improves Alzheimer’s disease-like pathologies and cognitive decline by promoting autophagy through activation of the AMPK/mTOR/ULK1 pathway. Biomed. Pharmacother..

[82] Liu G., Zhang T., Qian S., Zhang X., Lou H., Fan P. (2025). Conjugation with the XJB peptide enhanced neuroprotective effect of honokiol via SIRT3 modulation. Eur. J. Med. Chem..

[83] Li N., Liang Y., Zhang L., Xu C., Wang L. (2024). Neolignans in Magnolia officinalis as natural anti-Alzheimer’s disease agents: A systematic review. Ageing Res. Rev..

[84] Han R., Yu Y., Zhao K., Wei J., Hui Y., Gao J.M. (2022). Lignans from Eucommia ulmoides Oliver leaves exhibit neuroprotective effects via activation of the PI3K/Akt/GSK-3β/Nrf2 signaling pathways in H2O2-treated PC-12 cells. Phytomedicine.

[85] Senizza A., Rocchetti G., Mosele J.I., Patrone V., Callegari M.L., Morelli L., Lucini L. (2020). Lignans and Gut Microbiota: An Interplay Revealing Potential Health Implications. Molecules.

[86] Ghaderi S., Rashno M., Nesari A., Khoshnam S.E., Sarkaki A., Khorsandi L., Farbood Y., Rashidi K. (2021). Sesamin alleviates diabetes-associated behavioral deficits in rats: The role of inflammatory and neurotrophic factors. Int. Immunopharmacol..

[87] Zhao T.T., Shin K.S., Park H.J., Kim K.S., Lee K.E., Cho Y.J., Lee M.K. (2016). Effects of (-)-sesamin on chronic stress-induced memory deficits in mice. Neurosci. Lett..

[88] Cho Y.S., Song W.S., Yoon S.H., Park K.Y., Kim M.H. (2018). Syringaresinol suppresses excitatory synaptic transmission and picrotoxin-induced epileptic activity in the hippocampus through presynaptic mechanisms. Neuropharmacology.

[89] Jiang Q., Chen J., Long X.B., Yao X.L., Zou X., Yang Y.P., Huang G.Y., Zhang H.Q. (2020). Phillyrin protects mice from traumatic brain injury by inhibiting the inflammation of microglia via PPARγ signaling pathway. Int. Immunopharmacol..

[90] Orabi M.A.A., Abdelhamid R.A., Elimam H., Elshaier Y.A.M.M., Ali A.A., Aldabaan N., Alhasaniah A.H., Refaey M.S. (2024). Furofuranoid-Type Lignans and Related Phenolics from Anisacanthus virgularis (Salisb.) Nees with Promising Anticholinesterase and Anti-Ageing Properties: A Study Supported by Molecular Modelling. Plants.

[91] Wang F., Wen H., Liu L., Aisa H.A., Xin X. (2025). A Pair of Epimers of Lignan Alleviate Neuroinflammatory Effects by Modulating iNOS/COX-2 and MAPK/NF-κB Signaling Pathways. Inflammation.

[92] Han Y.K., Vinh L.B., Lee K.S., Lee M.K., Lee K.Y. (2025). Aurantiosides A–D: New Furofuran Lignan Glucosides with Neuroprotective Activity from Osmanthus fragrans var. aurantiacus Enabled via a MS/MS-Based Molecular Networking Strategy. ACS Omega.

[93] Ma Q., Chen L., Wei R. (2021). Isolation and characterization of neuroprotective lignans from salted Aconiti lateralis Radix Praeparata. Biosci. Biotechnol. Biochem..

[94] Somani G., Nahire M., Parikh A., Mulik M., Ghumatkar P., Laddha K., Sathaye S. (2017). Neuroprotective effect of Cubebin: A dibenzylbutyrolactone lignan on scopolamine-induced amnesia in mice. Indian J. Med. Res..

[95] Liu Q., Wang J., Lin B., Cheng Z.Y., Bai M., Shi S., Huang X.X., Song S.J. (2019). Phenylpropanoids and lignans from Prunus tomentosa seeds as efficient β-amyloid (Aβ) aggregation inhibitors. Bioorg. Chem..

[96] Wang Y.X., Zhou L., Wang J., Lin B., Wang X.B., Huang X.X., Song S.J. (2018). Enantiomeric lignans with anti-β-amyloid aggregation activity from the twigs and leaves of Pithecellobium clypearia Benth. Bioorg. Chem..

[97] Bajpai V., Bajpai V.K., Alam M.B., Quan K.T., Kwon K.R., Ju M.K., Choi H.J., Lee J.S., Yoon J.I., Majumder R. (2017). Antioxidant efficacy and the upregulation of Nrf2-mediated HO-1 expression by (+)-lariciresinol, a lignan isolated from Rubia philippinensis, through the activation of p38. Sci. Rep..

[98] Cheng M.J., Wu M.D., Su Y.S., Chen I.S., Yuan G.F. (2012). Anti-inflammatory compounds from monascus pilosus-fermented rice. Phytochem. Lett..

[99] Chaves M.H., Roque N.F. (1997). Amides and lignanamides from Porcelia macrocarpa. Phytochemistry.

[100] Zhang J.X., Guan S.H., Feng R.H., Wang Y., Wu Z.Y., Zhang Y.B., Chen X.H., Bi K.S., Guo D.A. (2013). Neolignanamides, lignanamides, and other phenolic compounds from the root bark of Lycium chinense. J. Nat. Prod..

[101] Xie L.W., Atanasov A.G., Guo D.A., Malainer C., Zhang J.X., Zehl M., Guan S.H., Heiss E.H., Urban E., Dirsch V.M. (2014). Activity-guided isolation of NF-κB inhibitors and PPARγ agonists from the root bark of Lycium chinense miller. J. Ethnopharmacol..

[102] Yang F., Li H., Yang Y.Q., Hou Y., Liang D. (2022). Lignanamides from the stems of Piper hancei maxim. and their anti-inflammatory and cytotoxic activities. Fitoterapia.

[103] Zhuang X.C., Chen G.L., Liu Y., Zhang Y.L., Guo M.Q. (2021). New Lignanamides with antioxidant and anti-inflammatory activities screened out and identified from Warburgia ugandensis combining affinity ultrafiltration LC-MS with SOD and XOD enzymes. Antioxidants.

[104] Benkirane C., Ben Moumen A., Fauconnier M.L., Belhaj K., Abid M., Caid H.S., Elamrani A., Mansouri F. (2022). Bioactive compounds from hemp (Cannabis sativa L.) seeds: Optimization of phenolic antioxidant extraction using simplex lattice mixture design and HPLC-DAD/ESI-MS2 analysis. RSC Adv..

[105] Zhang B., Huang R., Hua J., Liang H., Pan Y., Dai L., Liang D., Wang H. (2016). Antitumor lignanamides from the aerial parts of Corydalis saxicola. Phytomedicine.

[106] Sakakibara I., Katsuhara T., Ikeya Y., Hayashi K., Mitsuhashi H. (1991). Cannabisin a, an arylnaphthalene lignanamide from fruits of cannabis sativa. Phytochemistry.

[107] Sakakibara I. (1991). Allergy inhibitors containing lignans.

[108] Zhou Y., Wang S., Lou H., Fan P. (2017). Chemical constituents of hemp (Cannabis sativa L.) seed with potential anti-neuroinflammatory activity. Phytochem. Lett..

[109] Wu N., Shen Q., Cai G., Zha Y. (2009). Identification and free radical scavenging activity of lignanamide extract from fructus cannabis of bama. Acta Chim. Sin..

[110] Xu P.W., Yuan X.F., Zhao B. (2023). Bioactive polyphenols separated from hemp seed shells ameliorate H_2_O_2_-induced oxidative stress injury in human umbilical vein endothelial cells. J. Food Sci..

[111] Choi H.S., Cho J.Y., Jin M.R., Lee Y.G., Kim S.J., Ham K.S., Moon J.H. (2016). Phenolics, acyl galactopyranosyl glycerol, and lignan amides from Tetragonia tetragonioides (pall.) Tuntze. Food Sci. Biotechnol..

[112] Yang Q., Ye G., Zhao W.M. (2007). Chemical constituents of Commelina communis Linn. Biochem. Syst. Ecol..

[113] Wu Y., Zheng C.-J., Qin L. (2013). Two new bis-alkaloids from the aerial part of Piper flaviflorum. Helv. Chim. Acta.

[114] Sun J., Gu Y.F., Su X.Q., Li M.M., Huo H.X., Zhang J., Zeng K.W., Zhang Q., Zhao Y.F., Li J. (2014). Anti-inflammatory lignanamides from the roots of Solanum melongena L. Fitoterapia.

[115] Li J.X., Shi Q., Xiong Q.B., Prasain J.K., Tezuka Y., Hareyama T., Wang Z.T., Tanaka K., Namba T., Kadota S. (1998). Tribulusamide a and B, new hepatoprotective lignanamides from the fruits of Tribulus terrestris: Indications of cytoprotective activity in murine hepatocyte culture. Planta Med..

[116] Ge F., Tang C., Ye Y. (2008). Lignanamides and sesquiterpenoids from stems of Mitrephora thorelii. Helv. Chim. Acta.

[117] Meerungrueang W., Panichayupakaranant P. (2016). A new antibacterial tetrahydronaphthalene lignanamide, foveolatamide, from the stems of Ficus foveolata. Nat. Prod. Commun..

[118] King R.R., Calhoun L.A. (2005). Characterization of cross-linked hydroxycinnamic acid amides isolated from potato common scab lesions. Phytochemistry.

[119] Bolleddula J., Fitch W.L., Vareed S.K., Nair M.G. (2012). Identification of metabolites in Withania somnifera fruits by liquid chromatography and high-resolution mass spectrometry. Rapid Commun. Mass Spectrom..

[120] Bolleddulla J., Fitch B., Vareed S.K. (2013). Abstracts, 44th western regional meeting of the American Chemical Society, Santa Clara, CA. Abstr. Pap. Am. Chem. Soc..

[121] Lajide L., Escoubas P., Mizutani J. (1995). Termite antifeedant activity in Xylopia aethiopica. Phytochemistry.

[122] Wang P., Zhao G.-W., Xia W., Han E.-J., Xiang L. (2014). A new flavonol C-glycoside and a rare bioactive lignanamide from Piper wallichii Miq. Hand.-Maz. Chin. J. Nat. Med..

[123] Sakakibara I. JP 02149545 A, 1990. https://www.j-platpat.inpit.go.jp.

[124] Seca A.M.L., Silva A.M.S., Silvestre A.J.D., Cavaleiro J.A.S., Domingues F.M.J., Pascoal-Neto C. (2001). Lignanamides and other phenolic constituents from the bark of kenaf. Hibiscus cannabinus. Phytochemistry.

[125] Li W. (2013). Method for extraction of alpha-glucosidase activity inhibitor from Lycium seed and its residue after extraction of Lycium oil and its application for preparation of drugs for treating diabetes mellitus, aids and tumor.

[126] Yoshihara T., Yamaguchi K., Takamatsu S., Sakamura S. (1981). A new lignanamide, grossamide, from bell pepper. Biosci. Biotechnol. Biochem..

[127] Yoshihara T., Yamaguchi K., Sakamura S. (1983). The relative configuration of grossamide and hordatines. Biosci. Biotechnol. Biochem..

[128] Santos L.P., Boaventura M.A., de Oliveira A.B., Cassady J.M. (1996). Grossamide and N-trans-caffeoyltyramine from Annona crassiflora seeds. Planta Med..

[129] Zhu L.H., Huang X.-S., Ye W.-C., Zhou G.-X. (2012). Study on chemical constituents of Alocasia macrorrhiza Schott. J. Chin. Pharm. Sci..

[130] Kembelo P.K., Tuenter E., Vanhove W., Katula H.B., Van Damme P., Pieters L. (2023). Phytochemical profiling by UPLC-ESI-QTOF-MS of Commelina africana, widely used in traditional medicine in DR Congo. South Afr. J. Bot..

[131] Zhang J., Guo S.J., Chan K.W., Tong E.P.S., Fu G.M., Mu Q.Z., Ip F.C.F., Ip N.Y. (2016). New lignans with neuroprotective activity from Adelostemma gracillimum. Phytochem. Lett..

[132] Wang X., Ma W., Wang Y., Ren F.C., Wang K.J., Li N. (2024). Norlignans and phenolics from Curculigo capitulata and their neuroprotection against glutamate-induced oxidative injury in SH-SY5Y cells. Molecules.

[133] Ma Q., Liu Y., Liu S.N., Li Y.X., Chen H.L. (2020). Structural elucidation and neuroprotective activities of lignans from the flower buds of Magnolia biondii Pamp. Z. Naturforsch. C.

[134] Gao K., Liu M.Y., Li Y., Wang L., Zhao C., Zhao X., Zhao J.Y., Ding Y., Tang H.F., Jia Y.Y. (2021). Lyciumamide a, a dimer of phenolic amide, protects against NMDA-induced neurotoxicity and potential mechanisms in vitro. J. Mol. Histol..

[135] Gao K., Ma D.W., Chen Y., Tian X.R., Lu Y.Y., Du X.Y., Tang H.F., Chen J.Z. (2015). Three new dimers and two monomers of phenolic amides from the fruits of Lycium barbarum and their antioxidant activities. J. Agric. Food Chem..

[136] Zhou M., Yuan F., Ruan H.L., Li J., Huang J.F., Liu S., Huang T.Y., Zhang Y.J., Liang Q. (2023). Neuroprotective neolignan glycosides from the pseudobulbs of Bletilla striata. Fitoterapia.

[137] Su T., Pu M.-C., Tang D.-K., Long J.-C., Yuan F.-Y., Yin A.-P., Wu S.-Q., Yin S., Tang G.-H. (2023). New benzofuran neolignans with neuroprotective activity from Phyllanthodendron breynioides. Nat. Prod. Res..

[138] Kay H.Y., Kim Y.W., Ryu D.H., Sung S.H., Hwang S.J., Kim S.G. (2011). Nrf2-mediated liver protection by sauchinone, an antioxidant lignan, from acetaminophen toxicity through the PKCδ-GSK3β pathway. Br. J. Pharmacol..

[139] Hu Y., Zhang M., Liu B.H., Tang Y.Y., Wang Z., Wang T., Zheng J.X., Zhang J.J. (2023). Honokiol prevents chronic cerebral hypoperfusion induced astrocyte A1 polarization to alleviate neurotoxicity by targeting SIRT3-STAT3 axis. Free Radic. Biol. Med..

[140] Li Z., Zheng Y., Liu K., Liang Y.D., Lu J., He J.M., Zeng J.H., Liu Q.S., Li S.T. (2023). Lignans as multi-targeted natural products in neurodegenerative diseases and depression: Recent perspectives. Phytother. Res..

[141] Ghaderi M.A., Ghasemi M., Khodadadi H., Farbood Y. (2023). The Role of Sesamin in Targeting Neurodegenerative Disorders: A Systematic Review. Mini Rev. Med. Chem..

[142] Song M., Zhang S.S., Yu W.Q., Fan X. (2024). Gomisin N rescues cognitive impairment of Alzheimer’s disease by targeting GSK3β and activating Nrf2 signaling pathway. Phytomedicine.

[143] Jia S., Liu Y., Zhang H., Li Y., Wang Y., Zhao L. (2024). Schisandrin A Alleviates Inflammation and Oxidative Stress in Aβ25-35-Induced Alzheimer’s Disease in Vitro Model. Actas Esp. Psiquiatr..

[144] Wang X., Hu D., Zhang L.J., Lian G.N., Zhao S.Q., Wang C.M., Yin J., Wu C.F., Yang J.Y. (2014). Gomisin A inhibits lipopolysaccharide-induced inflammatory responses in N9 microglia via blocking the NF-κB/MAPKs pathway. Food Chem. Toxicol..

[145] Guo X., Lei M., Ma G.D., Ouyang C.H., Yang X.S., Liu C., Chen Q.J., Liu X.F. (2024). Schisandrin A Alleviates Spatial Learning and Memory Impairment in Diabetic Rats by Inhibiting Inflammatory Response and Through Modulation of the PI3K/AKT Pathway. Mol. Neurobiol..

[146] Ding T., Song M., Wu Y., Li Z., Zhang S., Fan X. (2025). Schisandrin B ameliorates Alzheimer’s disease by suppressing neuronal ferroptosis and ensuing microglia M1 polarization. Phytomedicine.

[147] Luo Q., Ji M., Yuan H.Q., Lou H.X., Fan P.H. (2017). Anti-neuroinflammatory effects of grossamide from hemp seed via suppression of TLR-4-mediated NF-κB signaling pathways in lipopolysaccharide-stimulated BV2 microglia cells. Mol. Cell. Biochem..

[148] Giridharan V.V., Thandavarayan R.A., Sato S., Ko K.M., Konishi T. (2011). Prevention of scopolamine-induced memory deficits by schisandrin B, an antioxidant lignan from Schisandra chinensis in mice. Free Radic. Res..

[149] Xi Y.F., Bai M., Zhang X., Hou Z.L., Lin B., Yao G.D., Lou L.L., Song S.J., Huang X.X. (2023). Insight into tetrahydrofuran lignans from Isatis indigotica fortune with neuroprotective and acetylcholinesterase inhibitor activity. Phytochemistry.

[150] Qi Y., Dou D.Q., Jiang H., Zhang B.B., Qin W.Y., Kang K., Zhang N., Jia D. (2017). Arctigenin Attenuates Learning and Memory Deficits through PI3k/Akt/GSK-3β Pathway Reducing Tau Hyperphosphorylation in Aβ-Induced AD Mice. Planta Med..

[151] Li H., Wang W., Hou T.T., Tian Y.R., Wu Q.Q., Xu L.Z., Wei Y.P., Wang X., Jia J.P. (2018). Honokiol Alleviates Cognitive Deficits of Alzheimer’s Disease (PS1V97L) Transgenic Mice by Activating Mitochondrial SIRT3. J. Alzheimers Dis..

[152] Zhou Y., Wang Y., Li Y., Wang Y., Zhang Y., Liu X., Liu Y. (2021). Pharmacokinetic and metabolomics approach based on UHPLC-MS to evaluate therapeutic effect of lignans from S. Chinensis in alzheimer’s disease. J. Chromatogr. B.

[153] Ehambarampillai D., Wan M.L.Y. (2025). A comprehensive review of Schisandra chinensis lignans: Pharmacokinetics, pharmacological mechanisms, and future prospects in disease prevention and treatment. Chin. Med..

[154] Wei M.Y., Liu Y., Pi Z., Li S., Hu M., He Y., Yue K., Liu T., Liu Z., Song F. (2019). Systematically characterize the anti-Alzheimer’s disease mechanism of lignans from S. chinensis based on in-vivo ingredient analysis and target-network pharmacology strategy by UHPLC-Q-TOF-MS. Molecules.

[155] Zhang F., Zhai J.X., Weng N., Gao J., Yin J., Chen W.S. (2022). A comprehensive review of the main lignan components of Schisandra chinensis (North Wu Wei Zi) and Schisandra sphenanthera (South Wu Wei Zi) and the lignan-induced drug-drug interactions based on the inhibition of cytochrome P450 and P-glycoprotein activities. Front. Pharmacol..

